# Supplementary material for: PsychRNN: An Accessible and Flexible Python Package for Training Recurrent Neural Network Models on Cognitive Tasks
Source: eNeuro. 2021 Jan 5;8(1):ENEURO.0427-20.2020. doi: 10.1523/ENEURO.0427-20.2020 (PMC7814477; doi:10.1523/ENEURO.0427-20.2020)
Supplement: Extended Data 1 — PsychRNN package and documentation. Download Extended Data 1, ZIP file. [file enu-eN-OTM-0427-20-s06.zip › PsychRNN Extended Data 1/docs/_build/html/_modules/psychrnn/tasks/perceptual_discrimination.html]

  


psychrnn.tasks.perceptual\_discrimination — PsychRNN 1.0.0-alpha documentation


PsychRNN

1.0

Contents:

- Installation Guide
- API Documentation
- Getting Started

PsychRNN

- Docs »
- Module code »
- psychrnn.tasks.perceptual\_discrimination

---

# Source code for psychrnn.tasks.perceptual\_discrimination

```
from __future__ import division

from psychrnn.tasks.task import Task
import numpy as np

[docs]class PerceptualDiscrimination(Task):
    """Two alternative forced choice (2AFC) binary discrimination task. 

    On each trial the network receives two simultaneous noisy inputs into each of two input channels. The network must determine which channel has the higher mean input and respond by driving the corresponding output unit to 1.

    Takes two channels of noisy input (:attr:`N_in` = 2).
    Two channel output (:attr:`N_out` = 2) with a one hot encoding (high value is 1, low value is .2) towards the higher mean channel.

    Loosely based on `Britten, Kenneth H., et al. "The analysis of visual motion: a comparison of neuronal and psychophysical performance." Journal of Neuroscience 12.12 (1992): 4745-4765 <https://www.jneurosci.org/content/12/12/4745>`_

    Args:
        dt (float): The simulation timestep.
        tau (float): The intrinsic time constant of neural state decay.
        T (float): The trial length.
        N_batch (int): The number of trials per training update.
        coherence (float, optional): Amount by which the means of the two channels will differ. By default None.
        direction (int, optional): Either 0 or 1, indicates which input channel will have higher mean input. By default None.

    """

    def __init__(self, dt, tau, T, N_batch, coherence = None, direction = None):
        super(PerceptualDiscrimination,self).__init__(2, 2, dt, tau, T, N_batch)
        
        self.coherence = coherence

        self.direction = direction

        self.lo = 0.2 # Low value for one hot encoding

        self.hi = 1.0 # High value for one hot encoding

[docs]    def generate_trial_params(self, batch, trial):
        """Define parameters for each trial.

        Implements :func:`~psychrnn.tasks.task.Task.generate_trial_params`.

        Args:
            batch (int): The batch number that this trial is part of.
            trial (int): The trial number of the trial within the batch *batch*.

        Returns:
            dict: Dictionary of trial parameters including the following keys:

            :Dictionary Keys: 
                * **coherence** (*float*) -- Amount by which the means of the two channels will differ. :attr:`self.coherence` if not None, otherwise ``np.random.exponential(scale=1/5)``.
                * **direction** (*int*) -- Either 0 or 1, indicates which input channel will have higher mean input. :attr:`self.direction` if not None, otherwise ``np.random.choice([0, 1])``.
                * **stim_noise** (*float*) -- Scales the stimlus noise. Set to .1.
                * **onset_time** (*float*) -- Stimulus onset time. ``np.random.random() * self.T / 2.0``.
                * **stim_duration** (*float*) -- Stimulus duration. ``np.random.random() * self.T / 4.0 + self.T / 8.0``.

        """

        # ----------------------------------
        # Define parameters of a trial
        # ----------------------------------
        params = dict()
        if self.coherence == None:
            params['coherence'] = np.random.choice([0.1, 0.3, 0.5, 0.7])
        else:
            params['coherence'] = self.coherence
        params['direction'] = np.random.choice([0, 1])
        params['stim_noise'] = 0.1
        params['onset_time'] = np.random.random() * self.T / 2.0
        params['stim_duration'] = np.random.random() * self.T / 4.0 + self.T / 8.0

        return params


[docs]    def trial_function(self, t, params):
        """Compute the trial properties at :data:`time`.

        Implements :func:`~psychrnn.tasks.task.Task.trial_function`.

        Based on the :data:`params` compute the trial stimulus (x_t), correct output (y_t), and mask (mask_t) at :data:`time`.

        Args:
            time (int): The time within the trial (0 <= :data:`time` < :attr:`T`).
            params (dict): The trial params produced by :func:`generate_trial_params`.

        Returns:
            tuple:

            * **x_t** (*ndarray(dtype=float, shape=(*:attr:`N_in` *,))*) -- Trial input at :data:`time` given :data:`params`. For ``params['onset_time'] < time < params['onset_time'] + params['stim_duration']`` , 1 is added to the noise in both channels, and :data:`params['coherence']` is also added in the channel corresponding to :data:`params[dir]`.
            * **y_t** (*ndarray(dtype=float, shape=(*:attr:`N_out` *,))*) -- Correct trial output at :data:`time` given :data:`params`. From ``time > params['onset_time'] + params[stim_duration] + 20`` onwards, the correct output is encoded using one-hot encoding. Until then, y_t is 0 in both channels.
            * **mask_t** (*ndarray(dtype=bool, shape=(*:attr:`N_out` *,))*) -- True if the network should train to match the y_t, False if the network should ignore y_t when training. The mask is True for ``time > params['onset_time'] + params['stim_duration']`` and False otherwise.

        """

        # ----------------------------------
        # Initialize with noise
        # ----------------------------------
        x_t = np.sqrt(2*.01*np.sqrt(10)*np.sqrt(self.dt)*params['stim_noise']*params['stim_noise'])*np.random.randn(self.N_in)
        y_t = np.zeros(self.N_out)
        mask_t = np.ones(self.N_out)

        # ----------------------------------
        # Retrieve parameters
        # ----------------------------------
        coh = params['coherence']
        onset = params['onset_time']
        stim_dur = params['stim_duration']
        dir = params['direction']

        # ----------------------------------
        # Compute values
        # ----------------------------------
        if onset < t < onset + stim_dur:
            x_t[dir] += 1 + coh
            x_t[(dir + 1) % 2] += 1

        if t > onset + stim_dur + 20:
            y_t[dir] = self.hi
            y_t[1-dir] = self.lo

        if t < onset + stim_dur:
            mask_t = np.zeros(self.N_out)

        return x_t, y_t, mask_t


[docs]    def accuracy_function(self, correct_output, test_output, output_mask):
        """Calculates the accuracy of :data:`test_output`.

        Implements :func:`~psychrnn.tasks.task.Task.accuracy_function`.

        Takes the channel-wise mean of the masked output for each trial. Whichever channel has a greater mean is considered to be the network's "choice".

        Returns:
            float: 0 <= accuracy <= 1. Accuracy is equal to the ratio of trials in which the network made the correct choice as defined above.
        
        """


        chosen = np.argmax(np.mean(test_output*output_mask, axis=1), axis = 1)
        truth = np.argmax(np.mean(correct_output*output_mask, axis = 1), axis = 1)
        return np.mean(np.equal(truth, chosen))
```

---

© Copyright 2020, Authors redacted for double-blind review

Built with Sphinx using a theme provided by Read the Docs.
