# Supplementary material for: PsychRNN: An Accessible and Flexible Python Package for Training Recurrent Neural Network Models on Cognitive Tasks
Source: eNeuro. 2021 Jan 5;8(1):ENEURO.0427-20.2020. doi: 10.1523/ENEURO.0427-20.2020 (PMC7814477; doi:10.1523/ENEURO.0427-20.2020)
Supplement: Extended Data 1 — PsychRNN package and documentation. Download Extended Data 1, ZIP file. [file enu-eN-OTM-0427-20-s06.zip › PsychRNN Extended Data 1/docs/_build/html/_modules/psychrnn/tasks/match_to_category.html]

  


psychrnn.tasks.match\_to\_category — PsychRNN 1.0.0-alpha documentation


PsychRNN

1.0

Contents:

- Installation Guide
- API Documentation
- Getting Started

PsychRNN

- Docs »
- Module code »
- psychrnn.tasks.match\_to\_category

---

# Source code for psychrnn.tasks.match\_to\_category

```
from __future__ import division

from psychrnn.tasks.task import Task
import numpy as np

[docs]class MatchToCategory(Task):
    """ Multidirectional decision-making task.

    On each trial the network receives input from units representing different locations on a ring. Each input unit magnitude represents closeness to the angle of input. The network must determine which side of arbitrary category boundaries the input belongs to and respond accordingly.

    Takes :attr:`N_in` channels of noisy input arranged in a ring with gaussian signal around the ring centered at 0 at the stimulus angle.
    :attr:`N_out` channel output arranged as slices of a ring with a one hot encoding towards the correct category output based on the angular location of the gaussian input bump.

    Loosely based on `Freedman, David J., and John A. Assad. "Experience-dependent representation of visual categories in parietal cortex." Nature 443.7107 (2006): 85-88. <https://www.nature.com/articles/nature05078>`_

    Args:
        dt (float): The simulation timestep.
        tau (float): The intrinsic time constant of neural state decay.
        T (float): The trial length.
        N_batch (int): The number of trials per training update.
        N_in (int, optional): The number of network inputs. Defaults to 16.
        N_out (int, optional): The number of network outputs. Defaults to 2.

    """
    
    def __init__(self, dt, tau, T, N_batch, N_in=16, N_out=2):
        super(MatchToCategory,self).__init__(N_in, N_out, dt, tau, T, N_batch)
        
    def _gaussian_input(self,angle, scale = 1):
        """ Calculates angular gaussian pdf with mean at the :data:`angle` for N_in evenly arranged around the circumference of a unit circle.

        Args:
            angle (float): The angle on the input circle at which to center the gaussian.
            scale (float, optional): The scale of the gaussian function. Defaults to 1.

        Returns:
            ndarray(dtype=float, shape=(:attr:`N_in`,)): Normal pdf value at (angle - angle of N_in channel).

        """
        
        g = np.zeros(self.N_in)
        map_g = np.linspace(0,2*np.pi,self.N_in)
        for ii in range(self.N_in):

            # Center N_in around angle, and truncate so -pi <= effective angle <= pi
            effective_angle = map_g[ii] - angle
            if effective_angle > np.pi:
                effective_angle = -2*np.pi + effective_angle
            elif effective_angle < -np.pi:
                effective_angle = 2*np.pi + effective_angle

            x = effective_angle/scale

            g[ii] = np.exp(-x**2/2.0) / (np.sqrt(2*np.pi)*scale)
        return g

[docs]    def generate_trial_params(self, batch, trial):
        """Define parameters for each trial.

        Implements :func:`~psychrnn.tasks.task.Task.generate_trial_params`.

        Args:
            batch (int): The batch number that this trial is part of.
            trial (int): The trial number of the trial within the batch *batch*.

        Returns:
            dict: Dictionary of trial parameters including the following keys:

            :Dictionary Keys: 
                * **angle** (*float*) -- Angle at which to center the gaussian. Randomly selected.
                * **category** (*int*) -- Index of the N_out category channel that contains the :data:`angle`.
                * **onset_time** (*float*) -- Stimulus onset time. Set to 200.
                * **input_dur** (*float*) -- Stimulus duration. Set to 1000.
                * **output_dur** (*float*) -- Output duration. The time given to make a choice. Set to 800.
                * **stim_noise** (*float*) -- Scales the stimlus noise. Set to .1.

        """
        params = dict()

        params['angle'] = 2*np.pi*np.random.rand()
        params['category'] = int(params['angle']/(2*np.pi/self.N_out))
        params['onset_time'] = 200
        params['input_dur'] = 1000.
        params['output_dur'] = 800.
        params['stim_noise'] = .1

        return params


[docs]    def trial_function(self, t, params):
        """Compute the trial properties at :data:`time`.

        Implements :func:`~psychrnn.tasks.task.Task.trial_function`.

        Based on the :data:`params` compute the trial stimulus (x_t), correct output (y_t), and mask (mask_t) at :data:`time`.

        Args:
            time (int): The time within the trial (0 <= :data:`time` < :attr:`T`).
            params (dict): The trial params produced by :func:`generate_trial_params`.

        Returns:
            tuple:

            * **x_t** (*ndarray(dtype=float, shape=(*:attr:`N_in` *,))*) -- Trial input at :data:`time` given :data:`params`. For ``params['onset_time'] < time < params['onset_time'] + params['input_dur']`` , gaussian pdf with mean = angle and scale = 1 is added to each input channel based on the channel's angle.
            * **y_t** (*ndarray(dtype=float, shape=(*:attr:`N_out` *,))*) -- Correct trial output at :data:`time` given :data:`params`. 1 in the :data:`params['category']` output channel during the output period defined by :data:`params['output_dur']`, 0 otherwise.
            * **mask_t** (*ndarray(dtype=bool, shape=(*:attr:`N_out` *,))*) -- True if the network should train to match the y_t, False if the network should ignore y_t when training. True during the output period, False otherwise.

        """
        # ----------------------------------
        # Initialize with noise
        # ----------------------------------
        x_t = (1./(2*self.dt))*params['stim_noise']*np.random.randn(self.N_in)
        y_t = np.zeros(self.N_out)
        mask_t = np.zeros(self.N_out)

        # ----------------------------------
        # Retrieve parameters
        # ----------------------------------
        onset_time = params['onset_time']
        input_dur = params['input_dur']
        output_dur = params['output_dur']
        
        angle = params['angle']
        category = params['category']


        if onset_time <= t < onset_time + input_dur:
            x_t += self._gaussian_input(angle)

        if onset_time + input_dur <= t < onset_time + input_dur + output_dur:
            y_t[category] += 1.
            mask_t = np.ones(self.N_out)

        return x_t, y_t, mask_t


[docs]    def accuracy_function(self, correct_output, test_output, output_mask):
        """Calculates the accuracy of :data:`test_output`.

        Implements :func:`~psychrnn.tasks.task.Task.accuracy_function`.

        Takes the channel-wise mean of the masked output for each trial. Whichever channel has a greater mean is considered to be the network's "choice".

        Returns:
            float: 0 <= accuracy <= 1. Accuracy is equal to the ratio of trials in which the network made the correct choice as defined above.
        
        """
        chosen = np.argmax(np.mean(test_output*output_mask, axis=1), axis = 1)
        truth = np.argmax(np.mean(correct_output*output_mask, axis = 1), axis = 1)
        return np.mean(np.equal(truth, chosen))
```

---

© Copyright 2020, Authors redacted for double-blind review

Built with Sphinx using a theme provided by Read the Docs.
