# Supplementary material for: PsychRNN: An Accessible and Flexible Python Package for Training Recurrent Neural Network Models on Cognitive Tasks
Source: eNeuro. 2021 Jan 5;8(1):ENEURO.0427-20.2020. doi: 10.1523/ENEURO.0427-20.2020 (PMC7814477; doi:10.1523/ENEURO.0427-20.2020)
Supplement: Extended Data 1 — PsychRNN package and documentation. Download Extended Data 1, ZIP file. [file enu-eN-OTM-0427-20-s06.zip › PsychRNN Extended Data 1/docs/_build/html/_modules/psychrnn/tasks/delayed_discrim.html]

  


psychrnn.tasks.delayed\_discrim — PsychRNN 1.0.0-alpha documentation


PsychRNN

1.0

Contents:

- Installation Guide
- API Documentation
- Getting Started

PsychRNN

- Docs »
- Module code »
- psychrnn.tasks.delayed\_discrim

---

# Source code for psychrnn.tasks.delayed\_discrim

```
from __future__ import division

from psychrnn.tasks.task import Task
import numpy as np

[docs]class DelayedDiscrimination(Task):
    """Delayed discrimination task. 

    Following a fore period, the network receives an input, followed by a delay. After the delay the network receives a second input. The second input channel receives noisy input that is inversely ordered compared to the input received by the first input channel. The network must respond by activating the output node that corresponds to the input channel with the greater input as the first stimulus.

    Takes two channels of noisy input (:attr:`N_in` = 2).
    Two channel output (:attr:`N_out` = 2) with a one hot encoding (high value is 1, low value is .2).

    Loosely based on `Romo, R., Brody, C. D., Hernández, A., & Lemus, L. (1999). Neuronal correlates of 
    parametric working memory in the prefrontal cortex. Nature, 399(6735), 470. <https://www.nature.com/articles/20939>`_

    Args:
        dt (float): The simulation timestep.
        tau (float): The intrinsic time constant of neural state decay.
        T (float): The trial length.
        N_batch (int): The number of trials per training update.
        onset_time (float, optional): Stimulus onset time in terms of trial length :data:`T`.
        stim_duration_1 (float, optional): Stimulus 1 duration in terms of trial length :data:`T`.
        delay_duration (float, optional): Delay duration in terms of trial length :data:`T`.
        stim_duration_2 (float, optional): Stimulus 2 duration in terms of trial length :data:`T`.
        decision_duration (float, optional): Decision duration  in terms of trial length :data:`T`.
    """

    def __init__(self, dt, tau, T, N_batch, onset_time = None, stim_duration_1 = None, delay_duration = None, stim_duration_2 = None, decision_duration = None):
        super(DelayedDiscrimination,self).__init__(2, 2, dt, tau, T, N_batch)
        
        self.onset_time = onset_time
        self.stim_duration_1 = stim_duration_1
        self.delay_duration = delay_duration
        self.stim_duration_2 = stim_duration_2
        self.decision_duration = decision_duration 

        self.frequency_pairs = [(18, 10), (22, 14), (26, 18), (30, 22), (34, 26)] # frequency pairs to select from
        self.decision_options = ['>', '<'] # decision options to select from

        self.lo = 0.2 # Low value for one hot encoding
        self.hi = 1.0 # High value for one hot encoding

    def _scale_p(self, f):
        """Scale frequency to be between .4 and 1.2."""
        return 0.4 + 0.8 * (f - 10) / (34 - 10)

    def _scale_n(self, f):
        """ Scale frequency to be between .4 and 1.2, invert frequency ordering."""
        return 0.4 + 0.8 * (34 - f) / (34 - 10)

[docs]    def generate_trial_params(self, batch, trial):
        """Define parameters for each trial.

        Implements :func:`~psychrnn.tasks.task.Task.generate_trial_params`.

        Args:
            batch (int): The batch number that this trial is part of.
            trial (int): The trial number of the trial within the batch *batch*.

        Returns:
            dict: Dictionary of trial parameters including the following keys:

            :Dictionary Keys: 
                * **stimulus_1** (*float*) -- Start time for stimulus one. :data:`onset_time`. 
                * **delay** (*float*) -- Start time for the delay. :data:`onset_time` + :data:`stimulus_duration_1`. 
                * **stimulus_2** (*float*) -- Start time in for stimulus one. :data:`onset_time` + :data:`stimulus_duration_1` + :data:`delay_duration`. 
                * **decision** (*float*) -- Start time in for decision period. :data:`onset_time` + :data:`stimulus_duration_1` + :data:`delay_duration` + :data:`stimulus_duration_2`. 
                * **end** (*float*) -- End of decision period. :data:`onset_time` + :data:`stimulus_duration_1` + :data:`delay_duration` + :data:`stimulus_duration_2` + :data:`decision_duration`. 
                * **stim_noise** (*float*) -- Scales the stimlus noise. Set to .1.
                * **f1** (*int*) -- Frequency of first stimulus. 
                * **f2** (*int*) -- Frequency of second stimulus.
                * **choice** (*str*) -- Indicates whether :data:`f1` is '>' or '<' :data:`f2`.
        """

        params = dict()

        if self.onset_time is None:
            onset_time = np.random.uniform(0, 1) * self.T / 8.0
        else:
            onset_time = self.onset_time
        
        if self.stim_duration_1 is None:
            stim_duration_1 = np.random.uniform(0, 1) * self.T / 4.0
        else:
            stim_duration_1 = self.stim_duration_1
        
        if self.delay_duration is None:
            delay_duration = np.random.uniform(0, 1) * self.T / 4.0
        else:
            delay_duration = self.delay_duration
        
        if self.stim_duration_2 is None:
            stim_duration_2 = np.random.uniform(0, 1) * self.T / 4.0
        else:
            stim_duration_2 = self.stim_duration_2
       
        if self.decision_duration is None:
            decision_duration = np.random.uniform(0, 1) * self.T / 8.0
        else:
            decision_duration = self.decision_duration
        
        params['stimulus_1'] = onset_time
        params['delay'] = onset_time + stim_duration_1
        params['stimulus_2'] = onset_time + stim_duration_1 + delay_duration
        params['decision'] = onset_time + stim_duration_1 + delay_duration + stim_duration_2
        params['end'] = onset_time + stim_duration_1 + delay_duration + stim_duration_2 + decision_duration

        params['stim_noise'] = 0.1

        fpair = self.frequency_pairs[np.random.choice(len(self.frequency_pairs))]
        gt_lt = np.random.choice(self.decision_options)
        if gt_lt == '>':
            f1, f2 = fpair
            choice = 0
        else:
            f2, f1 = fpair
            choice = 1
        params['f1'] = f1
        params['f2'] = f2
        params['choice'] = choice

        return params


[docs]    def trial_function(self, t, params):
        """Compute the trial properties at :data:`time`.

        Implements :func:`~psychrnn.tasks.task.Task.trial_function`.

        Based on the :data:`params` compute the trial stimulus (x_t), correct output (y_t), and mask (mask_t) at :data:`time`.

        Args:
            time (int): The time within the trial (0 <= :data:`time` < :attr:`T`).
            params (dict): The trial params produced by :func:`generate_trial_params`.

        Returns:
            tuple:

            * **x_t** (*ndarray(dtype=float, shape=(*:attr:`N_in` *,))*) -- Trial input at :data:`time` given :data:`params`. First channel contains :data:`f1` during the first stimulus period, and :data:`f2` during the second stimulus period, scaled to be between .4 and 1.2. Second channel contains the frequencies but reverse scaled -- high frequencies correspond to low values and vice versa. Both channels have baseline noise.
            * **y_t** (*ndarray(dtype=float, shape=(*:attr:`N_out` *,))*) -- Correct trial output at :data:`time` given :data:`params`. The correct output is encoded using one-hot encoding during the decision period.
            * **mask_t** (*ndarray(dtype=bool, shape=(*:attr:`N_out` *,))*) -- True if the network should train to match the y_t, False if the network should ignore y_t when training. The mask is True for during the decision period and False otherwise.
        
        """
        # ----------------------------------
        # Initialize with noise
        # ----------------------------------
        x_t = np.sqrt(2*.01*np.sqrt(10)*np.sqrt(self.dt)*params['stim_noise']*params['stim_noise'])*np.random.randn(self.N_in)
        y_t = np.zeros(self.N_out)
        mask_t = np.zeros(self.N_out)

        # ----------------------------------
        # Retrieve parameters
        # ----------------------------------
        stimulus_1 = params['stimulus_1']
        delay = params['delay']
        stimulus_2 = params['stimulus_2']
        decision = params['decision']
        end = params['end']

        f1 = params['f1']
        f2 = params['f2']
        choice = params['choice']

        # ----------------------------------
        # Compute values
        # ----------------------------------

        if stimulus_1 <= t < delay:
            x_t[0] += self._scale_p(f1)
            x_t[1] += self._scale_n(f1)

        if stimulus_2 <= t < decision:
            x_t[0] += self._scale_p(f2)
            x_t[1] += self._scale_n(f2)

        if decision <= t < end:
            y_t[choice] = self.hi
            y_t[1-choice] = self.lo
            mask_t = np.ones(self.N_out)

        return x_t, y_t, mask_t


[docs]    def accuracy_function(self, correct_output, test_output, output_mask):
        """Calculates the accuracy of :data:`test_output`.

        Implements :func:`~psychrnn.tasks.task.Task.accuracy_function`.

        Takes the channel-wise mean of the masked output for each trial. Whichever channel has a greater mean is considered to be the network's "choice".

        Returns:
            float: 0 <= accuracy <= 1. Accuracy is equal to the ratio of trials in which the network made the correct choice as defined above.
        
        """
        
        chosen = np.argmax(np.mean(test_output*output_mask, axis=1), axis = 1)
        truth = np.argmax(np.mean(correct_output*output_mask, axis = 1), axis = 1)
        return np.mean(np.equal(truth, chosen))
```

---

© Copyright 2020, Authors redacted for double-blind review

Built with Sphinx using a theme provided by Read the Docs.
