# Supplementary material for: PsychRNN: An Accessible and Flexible Python Package for Training Recurrent Neural Network Models on Cognitive Tasks
Source: eNeuro. 2021 Jan 5;8(1):ENEURO.0427-20.2020. doi: 10.1523/ENEURO.0427-20.2020 (PMC7814477; doi:10.1523/ENEURO.0427-20.2020)
Supplement: Extended Data 1 — PsychRNN package and documentation. Download Extended Data 1, ZIP file. [file enu-eN-OTM-0427-20-s06.zip › PsychRNN Extended Data 1/docs/_build/html/_modules/psychrnn/tasks/task.html]

  


psychrnn.tasks.task — PsychRNN 1.0.0-alpha documentation


PsychRNN

1.0

Contents:

- Installation Guide
- API Documentation
- Getting Started

PsychRNN

- Docs »
- Module code »
- psychrnn.tasks.task

---

# Source code for psychrnn.tasks.task

```
from __future__ import division

import numpy as np

from abc import ABCMeta, abstractmethod

# abstract class python 2 & 3 compatible
ABC = ABCMeta('ABC', (object,), {})

[docs]class Task(ABC):
    """ The base task class.

    The base task class provides the structure that users can use to\
    define a new task. This structure is used by example tasks \
    :class:`~psychrnn.tasks.perceptual_discrimination.PerceptualDiscrimination`, \
    :class:`~psychrnn.tasks.match_to_category.MatchToCategory`, \
    and :class:`~psychrnn.tasks.delayed_discrim.DelayedDiscrimination`.

    Note:
        The base task class is not itself a functioning task. 
        The generate_trial_params and trial_function must be defined to define a new, functioning, task.

    Args:
        N_in (int): The number of network inputs.
        N_out (int): The number of network outputs.
        dt (float): The simulation timestep.
        tau (float): The intrinsic time constant of neural state decay.
        T (float): The trial length.
        N_batch (int): The number of trials per training update.

    Inferred Parameters:
        * **alpha** (*float*) -- The number of unit time constants per simulation timestep.
        * **N_steps** (*int*): The number of simulation timesteps in a trial. 

    """
    def __init__(self, N_in, N_out, dt, tau, T, N_batch):

        # ----------------------------------
        # Initialize required parameters
        # ----------------------------------
        self.N_batch = N_batch
        self.N_in = N_in
        self.N_out = N_out
        self.dt = dt
        self.tau = tau
        self.T = T

        # ----------------------------------
        # Calculate implied parameters
        # ----------------------------------
        self.alpha = (1.0 * self.dt) / self.tau
        self.N_steps = int(np.ceil(self.T / self.dt))

[docs]    def get_task_params(self):
        """ Get dictionary of task parameters.

        Note:
            N_in, N_out, N_batch, dt, tau and N_steps must all be passed to the network model as parameters -- this function is the recommended way to begin building the network_params that will be passed into the RNN model.


        Returns:
            dict: Dictionary of :class:`Task` attributes including the following keys:

            :Dictionary Keys: 
                * **N_batch** (*int*) -- The number of trials per training update.
                * **N_in** (*int*) -- The number of network inputs.
                * **N_out** (*int*) -- The number of network outputs.
                * **dt** (*float*) -- The simulation timestep.
                * **tau** (*float*) -- The unit time constant.
                * **T** (*float*) -- The trial length.
                * **alpha** (*float*) -- The number of unit time constants per simulation timestep.
                * **N_steps** (*int*): The number of simulation timesteps in a trial. 

            Note:
                The dictionary will also include any other attributes defined in your task definition.
        
        """
        return self.__dict__


[docs]    @abstractmethod
    def generate_trial_params(self, batch, trial):
        """ Define parameters for each trial.

        Using a combination of randomness, presets, and task attributes, define the necessary trial parameters.

        Args:
            batch (int): The batch number for this trial.
            trial (int): The trial number of the trial within the batch data:`batch`.

        Returns:
            dict: Dictionary of trial parameters.


        Warning:
            This function is abstract and must be implemented in a child Task object.

        Example:
            See :func:`PerceptualDiscrimination <psychrnn.tasks.perceptual_discrimination.PerceptualDiscrimination.generate_trial_params>`,\
            :func:`MatchToCategory <psychrnn.tasks.match_to_category.MatchToCategory.generate_trial_params>`,\
            and :func:`DelayedDiscrimination <psychrnn.tasks.delayed_discrim.DelayedDiscrimination.generate_trial_params>` for example implementations.

        """
        pass


[docs]    @abstractmethod
    def trial_function(self, time, params):
        """ Compute the trial properties at :data:`time`.

        Based on the :data:'params' compute the trial stimulus (x_t), correct output (y_t), and mask (mask_t) at :data:`time`.

        Args:
            time (int): The time within the trial (0 <= :data:`time` < :attr:`T`).
            params (dict): The trial params produced by :func:`~psychrnn.tasks.task.Task.generate_trial_params`

        Returns:
            tuple:

            * **x_t** (*ndarray(dtype=float, shape=(*:attr:`N_in` *,))*) -- Trial input at :data:`time` given :data:`params`.
            * **y_t** (*ndarray(dtype=float, shape=(*:attr:`N_out` *,))*) -- Correct trial output at :data:`time` given :data:`params`.
            * **mask_t** (*ndarray(dtype=bool, shape=(*:attr:`N_out` *,))*) -- True if the network should train to match the y_t, False if the network should ignore y_t when training.

        Warning:
            This function is abstract and must be implemented in a child Task object.

        Example:
            See :func:`PerceptualDiscrimination <psychrnn.tasks.perceptual_discrimination.PerceptualDiscrimination.trial_function>`,\
            :func:`MatchToCategory <psychrnn.tasks.match_to_category.MatchToCategory.trial_function>`,\
            and :func:`DelayedDiscrimination <psychrnn.tasks.delayed_discrim.DelayedDiscrimination.trial_function>` for example implementations.
        
        """
        pass


[docs]    def accuracy_function(self, correct_output, test_output, output_mask):
        """ Function to calculate accuracy (not loss) as it would be measured experimentally.

        Output should range from 0 to 1. This function is used by :class:`~psychrnn.backend.curriculum.Curriculum` as part of it's :func:`~psychrnn.backend.curriculum.default_metric`.

        Args:
            correct_output(ndarray(dtype=float, shape =(:attr:`N_batch`, :attr:`N_steps`, :attr:`N_out` ))): Correct batch output. ``y_data`` as returned by :func:`batch_generator`.
            test_output(ndarray(dtype=float, shape =(:attr:`N_batch`, :attr:`N_steps`, :attr:`N_out` ))): Output to compute the accuracy of. ``output`` as returned by :func:`psychrnn.backend.rnn.RNN.test`.
            output_mask(ndarray(dtype=bool, shape =(:attr:`N_batch`, :attr:`N_steps`, :attr:`N_out`))): Mask. ``mask`` as returned by func:`batch_generator`.

        Returns:
            float: 0 <= accuracy <=1

        Warning:
            This function is abstract and may optionally be implemented in a child Task object.

        Example:
            See :func:`PerceptualDiscrimination <psychrnn.tasks.perceptual_discrimination.PerceptualDiscrimination.accuracy_function>`,\
            :func:`MatchToCategory <psychrnn.tasks.match_to_category.MatchToCategory.accuracy_function>`,\
            and :func:`DelayedDiscrimination <psychrnn.tasks.delayed_discrim.DelayedDiscrimination.accuracy_function>` for example implementations.
        """
        pass


[docs]    def generate_trial(self, params):
        """ Loop to generate a single trial.

        Args:
            params(dict): Dictionary of trial parameters generated by :func:`generate_trial_params`.

        Returns:
            tuple:

            * **x_trial** (*ndarray(dtype=float, shape=(*:attr:`N_steps`, :attr:`N_in` *))*) -- Trial input given :data:`params`.
            * **y_trial** (*ndarray(dtype=float, shape=(*:attr:`N_steps`, :attr:`N_out` *))*) -- Correct trial output given :data:`params`.
            * **mask_trial** (*ndarray(dtype=bool, shape=(*:attr:`N_steps`, :attr:`N_out` *))*) -- True during steps where the network should train to match :data:`y`, False where the network should ignore :data:`y` during training.
        """

        # ----------------------------------
        # Loop to generate a single trial
        # ----------------------------------
        x_data = np.zeros([self.N_steps, self.N_in])
        y_data = np.zeros([self.N_steps, self.N_out])
        mask = np.zeros([self.N_steps, self.N_out])

        for t in range(self.N_steps):
            x_data[t, :], y_data[t, :], mask[t, :] = self.trial_function(t * self.dt, params)

        return x_data, y_data, mask


[docs]    def batch_generator(self):
        """ Generates a batch of trials.

        Returns:
            Generator[tuple, None, None]:

        Yields:
            tuple:

            * **stimulus** (*ndarray(dtype=float, shape =(*:attr:`N_batch`, :attr:`N_steps`, :attr:`N_out` *))*): Task stimuli for :attr:`N_batch` trials.
            * **target_output** (*ndarray(dtype=float, shape =(*:attr:`N_batch`, :attr:`N_steps`, :attr:`N_out` *))*): Target output for the network on :attr:`N_batch` trials given the :data:`stimulus`.
            * **output_mask** (*ndarray(dtype=bool, shape =(*:attr:`N_batch`, :attr:`N_steps`, :attr:`N_out` *))*): Output mask for :attr:`N_batch` trials. True when the network should aim to match the target output, False when the target output can be ignored.
            * **trial_params** (*ndarray(dtype=dict, shape =(*:attr:`N_batch` *,))*): Array of dictionaries containing the trial parameters produced by :func:`generate_trial_params` for each trial in :attr:`N_batch`.
        
        """

        batch = 1
        while batch > 0:

            x_data = []
            y_data = []
            mask = []
            params = []
            # ----------------------------------
            # Loop over trials in batch
            # ----------------------------------
            for trial in range(self.N_batch):
                # ---------------------------------------
                # Generate each trial based on its params
                # ---------------------------------------
                p = self.generate_trial_params(batch, trial)
                x,y,m = self.generate_trial(p)
                x_data.append(x)
                y_data.append(y)
                mask.append(m)
                params.append(p)

            batch += 1

            yield np.array(x_data), np.array(y_data), np.array(mask), np.array(params)


[docs]    def get_trial_batch(self):
        """Get a batch of trials.

        Wrapper for :code:`next(self.batch_generator())`.

        Returns:
            tuple:

            * **stimulus** (*ndarray(dtype=float, shape =(*:attr:`N_batch`, :attr:`N_steps`, :attr:`N_in` *))*): Task stimuli for :attr:`N_batch` trials.
            * **target_output** (*ndarray(dtype=float, shape =(*:attr:`N_batch`, :attr:`N_steps`, :attr:`N_out` *))*): Target output for the network on :attr:`N_batch` trials given the :data:`stimulus`.
            * **output_mask** (*ndarray(dtype=bool, shape =(*:attr:`N_batch`, :attr:`N_steps`, :attr:`N_out` *))*): Output mask for :attr:`N_batch` trials. True when the network should aim to match the target output, False when the target output can be ignored.
            * **trial_params** (*ndarray(dtype=dict, shape =(*:attr:`N_batch` *,))*): Array of dictionaries containing the trial parameters produced by :func:`generate_trial_params` for each trial in :attr:`N_batch`.

        """
        return next(self.batch_generator())
```

---

© Copyright 2020, Authors redacted for double-blind review

Built with Sphinx using a theme provided by Read the Docs.
