# Supplementary material for: PsychRNN: An Accessible and Flexible Python Package for Training Recurrent Neural Network Models on Cognitive Tasks
Source: eNeuro. 2021 Jan 5;8(1):ENEURO.0427-20.2020. doi: 10.1523/ENEURO.0427-20.2020 (PMC7814477; doi:10.1523/ENEURO.0427-20.2020)
Supplement: Extended Data 1 — PsychRNN package and documentation. Download Extended Data 1, ZIP file. [file enu-eN-OTM-0427-20-s06.zip › PsychRNN Extended Data 1/docs/_build/html/_modules/psychrnn/backend/rnn.html]

  


psychrnn.backend.rnn — PsychRNN 1.0.0-alpha documentation


PsychRNN

1.0

Contents:

- Installation Guide
- API Documentation
- Getting Started

PsychRNN

- Docs »
- Module code »
- psychrnn.backend.rnn

---

# Source code for psychrnn.backend.rnn

```
from __future__ import division
from __future__ import print_function

from abc import ABCMeta, abstractmethod

# abstract class python 2 & 3 compatible
ABC = ABCMeta('ABC', (object,), {})

import tensorflow as tf
import numpy as np

import sys
from time import time
from os import makedirs, path
from inspect import isgenerator

from psychrnn.backend.regularizations import Regularizer
from psychrnn.backend.loss_functions import LossFunction
from psychrnn.backend.initializations import WeightInitializer, GaussianSpectralRadius

tf.compat.v1.disable_eager_execution()

[docs]class RNN(ABC):
    """ The base recurrent neural network class.

    Note:
        The base RNN class is not itself a functioning RNN. 
        forward_pass must be implemented to define a functioning RNN.

    Args:
       params (dict): The RNN parameters. Use your tasks's :func:`~psychrnn.tasks.task.Task.get_task_params` function to start building this dictionary. Optionally use a different network's :func:`get_weights` function to initialize the network with preexisting weights.

       :Dictionary Keys: 
            * **name** (*str*) -- Unique name used to determine variable scope. Having different variable scopes allows multiple distinct models to be instantiated in the same TensorFlow environment. See `TensorFlow's variable_scope <https://www.tensorflow.org/api_docs/python/tf/compat/v1/variable_scope>`_ for more details.
            * **N_in** (*int*) -- The number of network inputs.
            * **N_rec** (*int*) -- The number of recurrent units in the network.
            * **N_out** (*int*) -- The number of network outputs.
            * **N_steps** (*int*): The number of simulation timesteps in a trial. 
            * **dt** (*float*) -- The simulation timestep.
            * **tau** (*float*) -- The intrinsic time constant of neural state decay.
            * **N_batch** (*int*) -- The number of trials per training update.
            * **rec_noise** (*float, optional*) -- How much recurrent noise to add each time the new state of the network is calculated. Default: 0.0.
            * **transfer_function** (*function, optional*) -- Transfer function to use for the network. Default: `tf.nn.relu <https://www.tensorflow.org/api_docs/python/tf/nn/relu>`_.
            * **load_weights_path** (*str, optional*) -- When given a path, loads weights from file in that path. Default: None
            * **initializer** (:class:`~psychrnn.backend.initializations.WeightInitializer` *or child object, optional*) -- Initializer to use for the network. Default: :class:`~psychrnn.backend.initializations.WeightInitializer` (:data:`params`) if :data:`params` includes :data:`W_rec` or :data:`load_weights_path` as a key, :class:`~psychrnn.backend.initializations.GaussianSpectralRadius` (:data:`params`) otherwise.
            * **W_in_train** (*bool, optional*) -- True if input weights, W_in, are trainable. Default: True
            * **W_rec_train** (*bool, optional*) -- True if recurrent weights, W_rec, are trainable. Default: True
            * **W_out_train** (*bool, optional*) -- True if output weights, W_out, are trainable. Default: True
            * **b_rec_train** (*bool, optional*) -- True if recurrent bias, b_rec, is trainable. Default: True
            * **b_out_train** (*bool, optional*) -- True if output bias, b_out, is trainable. Default: True
            * **init_state_train** (*bool, optional*) -- True if the inital state for the network, init_state, is trainable. Default: True
            * **loss_function** (*str, optional*) -- Which loss function to use. See :class:`psychrnn.backend.loss_functions.LossFunction` for details. Defaults to ``"mean_squared_error"``.


        :Other Dictionary Keys:
            * Any dictionary keys used by the regularizer will be passed onwards to :class:`psychrnn.backend.regularizations.Regularizer`. See :class:`~psychrnn.backend.regularizations.Regularizer` for key names and details.
            * Any dictionary keys used for the loss function will be passed onwards to :class:`psychrnn.backend.loss_functions.LossFunction`. See :class:`~psychrnn.backend.loss_functions.LossFunction` for key names and details.
            * If :data:`initializer` is not set, any dictionary keys used by the initializer will be pased onwards to :class:`WeightInitializer <psychrnn.backend.initializations.WeightInitializer>` if :data:`load_weights_path` is set or :data:`W_rec` is passed in. Otherwise all keys will be passed to :class:`GaussianSpectralRadius <psychrnn.backend.initializations.GaussianSpectralRadius>`
            * If :data:`initializer` is not set and :data:`load_weights_path` is not set, the dictionary entries returned previously by :func:`get_weights` can be passed in to initialize the network. See :class:`WeightInitializer <psychrnn.backend.initializations.WeightInitializer>` for a list and explanation of possible parameters. At a minimum, :data:`W_rec` must be included as a key to make use of this option.
            * If :data:`initializer` is not set and :data:`load_weights_path` is not set, the following keys can be used to set biological connectivity constraints:

                * **input_connectivity** (*ndarray(dtype=float, shape=(* :attr:`N_rec`, :attr:`N_in` *)), optional*) -- Connectivity mask for the input layer. 1 where connected, 0 where unconnected. Default: np.ones((:attr:`N_rec`, :attr:`N_in`)).
                * **rec_connectivity** (*ndarray(dtype=float, shape=(* :attr:`N_rec`, :attr:`N_rec` *)), optional*) -- Connectivity mask for the recurrent layer. 1 where connected, 0 where unconnected. Default: np.ones((:attr:`N_rec`, :attr:`N_rec`)).
                * **output_connectivity** (*ndarray(dtype=float, shape=(* :attr:`N_out`, :attr:`N_rec` *)), optional*) -- Connectivity mask for the output layer. 1 where connected, 0 where unconnected. Default: np.ones((:attr:`N_out`, :attr:`N_rec`)).
                * **autapses** (*bool, optional*) -- If False, self connections are not allowed in N_rec, and diagonal of :data:`rec_connectivity` will be set to 0. Default: True.
                * **dale_ratio** (float, optional) -- Dale's ratio, used to construct Dale_rec and Dale_out. 0 <= dale_ratio <=1 if dale_ratio should be used. ``dale_ratio * N_rec`` recurrent units will be excitatory, the rest will be inhibitory. Default: None
        
        Inferred Parameters:
            * **alpha** (*float*) -- The number of unit time constants per simulation timestep.

    """
    def __init__(self, params):
        self.params = params

        # --------------------------------------------
        # Unique name used to determine variable scope
        # --------------------------------------------
        try:
            self.name = params['name']
        except KeyError:
            print("You must pass a  'name' to RNN")
            raise

        # ----------------------------------
        # Network sizes (tensor dimensions)
        # ----------------------------------
        try:
            N_in = self.N_in = params['N_in']
        except KeyError:
            print("You must pass 'N_in' to RNN")
            raise
        try:
            N_rec = self.N_rec = params['N_rec']
        except KeyError:
            print("You must pass 'N_rec' to RNN")
            raise
        try:
            N_out = self.N_out = params['N_out']
        except KeyError:
            print("You must pass 'N_out' to RNN")
            raise
        try:
            N_steps = self.N_steps = params['N_steps']
        except KeyError:
            print("You must pass 'N_steps' to RNN")
            raise

        # ----------------------------------
        # Physical parameters
        # ----------------------------------
        try:
            self.dt = params['dt']
        except KeyError:
            print("You must pass 'dt' to RNN")
            raise
            
        try:
            self.tau = params['tau']
        except KeyError:
            print("You must pass 'tau' to RNN")
            raise
        try: 
            self.tau = self.tau.astype('float32')
        except AttributeError:
            pass

        try:
            self.N_batch = params['N_batch']
        except KeyError:
            print("You must pass 'N_batch' to RNN")
            raise
            
        self.alpha = (1.0 * self.dt) / self.tau
        self.rec_noise = params.get('rec_noise', 0.0)
        self.transfer_function = params.get('transfer_function', tf.nn.relu)


        # ----------------------------------
        # Load weights path
        # ----------------------------------
        self.load_weights_path = params.get('load_weights_path', None)

        # ------------------------------------------------
        # Define initializer for TensorFlow variables
        # ------------------------------------------------
        if self.load_weights_path is not None:
            self.initializer = WeightInitializer(load_weights_path=self.load_weights_path)
        elif params.get('W_rec', None) is not None:
            self.initializer = params.get('initializer',
                                          WeightInitializer(**params))
        else:
            self.initializer = params.get('initializer',
                                          GaussianSpectralRadius(**params))

        self.dale_ratio = self.initializer.get_dale_ratio()

        # ----------------------------------
        # Trainable features
        # ----------------------------------
        self.W_in_train = params.get('W_in_train', True)
        self.W_rec_train = params.get('W_rec_train', True)
        self.W_out_train = params.get('W_out_train', True)
        self.b_rec_train = params.get('b_rec_train', True)
        self.b_out_train = params.get('b_out_train', True)
        self.init_state_train = params.get('init_state_train', True)

        # --------------------------------------------------
        # TensorFlow input/output placeholder initializations
        # ---------------------------------------------------
        self.x = tf.compat.v1.placeholder("float", [None, N_steps, N_in])
        self.y = tf.compat.v1.placeholder("float", [None, N_steps, N_out])
        self.output_mask = tf.compat.v1.placeholder("float", [None, N_steps, N_out])

        # --------------------------------------------------
        # Initialize variables in proper scope
        # ---------------------------------------------------
        with tf.compat.v1.variable_scope(self.name) as scope:
            # ------------------------------------------------
            # Trainable variables:
            # Initial State, weight matrices and biases
            # ------------------------------------------------
            try:
                self.init_state = tf.compat.v1.get_variable('init_state', [1, N_rec],
                                              initializer=self.initializer.get('init_state'),
                                              trainable=self.init_state_train)
            except ValueError as error:
                raise UserWarning("Try calling model.destruct() or changing params['name'].")


            self.init_state = tf.tile(self.init_state, [self.N_batch, 1])

            # Input weight matrix:
            self.W_in = \
                tf.compat.v1.get_variable('W_in', [N_rec, N_in],
                                initializer=self.initializer.get('W_in'),
                                trainable=self.W_in_train)

            # Recurrent weight matrix:
            self.W_rec = \
                tf.compat.v1.get_variable(
                    'W_rec',
                    [N_rec, N_rec],
                    initializer=self.initializer.get('W_rec'),
                    trainable=self.W_rec_train)

            # Output weight matrix:
            self.W_out = tf.compat.v1.get_variable('W_out', [N_out, N_rec],
                                         initializer=self.initializer.get('W_out'),
                                         trainable=self.W_out_train)

            # Recurrent bias:
            self.b_rec = tf.compat.v1.get_variable('b_rec', [N_rec], initializer=self.initializer.get('b_rec'),
                                         trainable=self.b_rec_train)
            # Output bias:
            self.b_out = tf.compat.v1.get_variable('b_out', [N_out], initializer=self.initializer.get('b_out'),
                                         trainable=self.b_out_train)

            # ------------------------------------------------
            # Non-trainable variables:
            # Overall connectivity and Dale's law matrices
            # ------------------------------------------------

            # Recurrent Dale's law weight matrix:
            self.Dale_rec = tf.compat.v1.get_variable('Dale_rec', [N_rec, N_rec],
                                            initializer=self.initializer.get('Dale_rec'),
                                            trainable=False)

            # Output Dale's law weight matrix:
            self.Dale_out = tf.compat.v1.get_variable('Dale_out', [N_rec, N_rec],
                                            initializer=self.initializer.get('Dale_out'),
                                            trainable=False)

            # Connectivity weight matrices:
            self.input_connectivity = tf.compat.v1.get_variable('input_connectivity', [N_rec, N_in],
                                                      initializer=self.initializer.get('input_connectivity'),
                                                      trainable=False)
            self.rec_connectivity = tf.compat.v1.get_variable('rec_connectivity', [N_rec, N_rec],
                                                    initializer=self.initializer.get('rec_connectivity'),
                                                    trainable=False)
            self.output_connectivity = tf.compat.v1.get_variable('output_connectivity', [N_out, N_rec],
                                                       initializer=self.initializer.get('output_connectivity'),
                                                       trainable=False)

        # --------------------------------------------------
        # Flag to check if variables initialized, model built
        # ---------------------------------------------------
        self.is_initialized = False
        self.is_built = False

[docs]    def build(self):
        """ Build the TensorFlow network and start a TensorFlow session.

        """
        # --------------------------------------------------
        # Define the predictions
        # --------------------------------------------------
        self.predictions, self.states = self.forward_pass()

        # --------------------------------------------------
        # Define the loss (based on the predictions)
        # --------------------------------------------------
        self.loss = LossFunction(self.params).set_model_loss(self)

        # --------------------------------------------------
        # Define the regularization
        # --------------------------------------------------
        self.reg = Regularizer(self.params).set_model_regularization(self)

        # --------------------------------------------------
        # Define the total regularized loss
        # --------------------------------------------------
        self.reg_loss = self.loss + self.reg

        # --------------------------------------------------
        # Open a session
        # --------------------------------------------------
        self.sess = tf.compat.v1.Session()

        # --------------------------------------------------
        # Record successful build
        # --------------------------------------------------
        self.is_built = True

        return


[docs]    def destruct(self):
        """ Close the TensorFlow session and reset the global default graph.

        """
        # --------------------------------------------------
        # Close the session. Delete the graph.
        # --------------------------------------------------
        if self.is_built:
            self.sess.close()
        tf.compat.v1.reset_default_graph()
        return


[docs]    def get_effective_W_rec(self):
        """ Get the recurrent weights used in the network, after masking by connectivity and dale_ratio.

        Returns:
            tf.Tensor(dtype=float, shape=(:attr:`N_rec`, :attr:`N_rec` ))

        """
        W_rec = self.W_rec * self.rec_connectivity
        if self.dale_ratio:
            W_rec = tf.matmul(tf.abs(W_rec), self.Dale_rec, name="in_1")
        return W_rec


[docs]    def get_effective_W_in(self):
        """ Get the input weights used in the network, after masking by connectivity and dale_ratio.

        Returns:
            tf.Tensor(dtype=float, shape=(:attr:`N_rec`, :attr:`N_in` ))
        """

        W_in = self.W_in * self.input_connectivity
        if self.dale_ratio:
            W_in = tf.abs(W_in)
        return W_in


[docs]    def get_effective_W_out(self):
        """ Get the output weights used in the network, after masking by connectivity, and dale_ratio.

        Returns:
            tf.Tensor(dtype=float, shape=(:attr:`N_out`, :attr:`N_rec` ))
        """

        W_out = self.W_out * self.output_connectivity
        if self.dale_ratio:
            W_out = tf.matmul(tf.abs(self.W_out), self.Dale_out, name="in_2")
        return W_out


[docs]    @abstractmethod
    def forward_pass(self):
        """ Run the RNN on a batch of task inputs. 

        Note:
            This is an abstract function that must be defined in a child class.
        
        Returns: 
            tuple:
            * **predictions** (*ndarray(dtype=float, shape=(*:attr:`N_batch`, :attr:`N_steps`, :attr:`N_out` *))*) -- Network output on inputs found in self.x within the tf network.
            * **states** (*ndarray(dtype=float, shape=(*:attr:`N_batch`, :attr:`N_steps`, :attr:`N_rec` *))*) -- State variable values over the course of the trials found in self.x within the tf network.

        """
        raise UserWarning("forward_pass must be implemented in child class. See Basic for example.")


[docs]    def get_weights(self):
        """ Get weights used in the network. 

        Allows for rebuilding or tweaking different weights to do experiments / analyses.

        Returns:
            dict: Dictionary of rnn weights including the following keys:

            :Dictionary Keys: 
                * **init_state** (*ndarray(dtype=float, shape=(1, :attr:`N_rec` *))*) -- Initial state of the network's recurrent units.
                * **W_in** (*ndarray(dtype=float, shape=(:attr:`N_rec`. :attr:`N_in` *))*) -- Input weights.
                * **W_rec** (*ndarray(dtype=float, shape=(:attr:`N_rec`, :attr:`N_rec` *))*) -- Recurrent weights.
                * **W_out** (*ndarray(dtype=float, shape=(:attr:`N_out`, :attr:`N_rec` *))*) -- Output weights.
                * **b_rec** (*ndarray(dtype=float, shape=(:attr:`N_rec`, *))*) -- Recurrent bias.
                * **b_out** (*ndarray(dtype=float, shape=(:attr:`N_out`, *))*) -- Output bias.
                * **Dale_rec** (*ndarray(dtype=float, shape=(:attr:`N_rec`, :attr:`N_rec`*))*) -- Diagonal matrix with ones and negative ones on the diagonal. If :data:`dale_ratio` is not ``None``, indicates whether a recurrent unit is excitatory(1) or inhibitory(-1).
                * **Dale_out** (*ndarray(dtype=float, shape=(:attr:`N_rec`, :attr:`N_rec`*))*) -- Diagonal matrix with ones and zeroes on the diagonal. If :data:`dale_ratio` is not ``None``, indicates whether a recurrent unit is excitatory(1) or inhibitory(0). Inhibitory neurons do not contribute to the output.
                * **input_connectivity** (*ndarray(dtype=float, shape=(:attr:`N_rec`, :attr:`N_in`*))*) -- Connectivity mask for the input layer. 1 where connected, 0 where unconnected.
                * **rec_connectivity** (*ndarray(dtype=float, shape=(:attr:`N_rec`, :attr:`N_rec`*))*) -- Connectivity mask for the recurrent layer. 1 where connected, 0 where unconnected.
                * **output_connectivity** (*ndarray(dtype=float, shape=(:attr:`N_out`, :attr:`N_rec`*))*) -- Connectivity mask for the output layer. 1 where connected, 0 where unconnected.
                * **dale_ratio** (*float*) -- Dale's ratio, used to construct Dale_rec and Dale_out. Either ``None`` if dale's law was not applied, or 0 <= dale_ratio <=1 if dale_ratio was applied.

            Note:
                Keys returned may be different / include other keys depending on the implementation of :class:`RNN` used. A different set of keys will be included e.g. if the :class:`~psychrnn.backend.models.lstm.LSTM` implementation is used. The set of keys above is accurate and meaningful for the :class:`~psychrnn.backend.models.basic.Basic` and :class:`~psychrnn.backend.models.basic.BasicScan` implementations.
        """
        if not self.is_built:
            self.build()

        if not self.is_initialized:
            self.sess.run(tf.compat.v1.global_variables_initializer())
            self.is_initialized = True
      
        weights_dict = dict()
        
        for var in tf.compat.v1.get_collection(tf.compat.v1.GraphKeys.GLOBAL_VARIABLES, scope=self.name):
            # avoid saving duplicates
            if var.name.endswith(':0') and var.name.startswith(self.name):
                name = var.name[len(self.name)+1:-2]
                weights_dict.update({name: var.eval(session=self.sess)})
        weights_dict.update({'W_rec': self.get_effective_W_rec().eval(session=self.sess)})
        weights_dict.update({'W_in': self.get_effective_W_in().eval(session=self.sess)})
        weights_dict.update({'W_out': self.get_effective_W_out().eval(session=self.sess)})
        weights_dict['dale_ratio'] = self.dale_ratio
        return weights_dict


[docs]    def save(self, save_path):
        """ Save the weights returned by :func:`get_weights` to :data:`save_path`

        Arguments:
            save_path (str): Path for where to save the network weights.

        """

        weights_dict = self.get_weights()

        np.savez(save_path, **weights_dict)

        return


[docs]    def train(self, trial_batch_generator, train_params={}):
        """ Train the network.

        Arguments:
            trial_batch_generator (:class:`~psychrnn.tasks.task.Task` object or *Generator[tuple, None, None]*): the task to train on, or the task to train on's batch_generator. If a task is passed in, task.:func:`batch_generator` () will be called to get the generator for the task to train on.
            train_params (dict, optional): Dictionary of training parameters containing the following possible keys:

                :Dictionary Keys: 
                    * **learning_rate** (*float, optional*) -- Sets learning rate if use default optimizer Default: .001
                    * **training_iters** (*int, optional*) -- Number of iterations to train for Default: 50000.
                    * **loss_epoch** (*int, optional*) -- Compute and record loss every 'loss_epoch' epochs. Default: 10.
                    * **verbosity** (*bool, optional*) -- If true, prints information as training progresses. Default: True.
                    * **save_weights_path** (*str, optional*) -- Where to save the model after training. Default: None
                    * **save_training_weights_epoch** (*int, optional*) -- Save training weights every 'save_training_weights_epoch' epochs. Weights only actually saved if :data:`training_weights_path` is set. Default: 100.
                    * **training_weights_path** (*str, optional*) -- What directory to save training weights into as training progresses. Default: None.               
                    * **curriculum** (`~psychrnn.backend.curriculum.Curriculum` *object, optional*) -- Curriculum to train on. If a curriculum object is provided, it overrides the trial_batch_generator argument. Default: None.
                    * **optimizer** (`tf.compat.v1.train.Optimizer <https://www.tensorflow.org/api_docs/python/tf/compat/v1/train/Optimizer>`_ *object, optional*) -- What optimizer to use to compute gradients. Default: `tf.train.AdamOptimizer <https://www.tensorflow.org/api_docs/python/tf/compat/v1/train/AdamOptimizer>`_ (learning_rate=:data:`train_params`['learning_rate']` ).
                    * **clip_grads** (*bool, optional*) -- If true, clip gradients by norm 1. Default: True
                    * **fixed_weights** (*dict, optional*) -- By default all weights are allowed to train unless :data:`fixed_weights` or :data:`W_rec_train`, :data:`W_in_train`, or :data:`W_out_train` are set. Default: None. Dictionary of weights to fix (not allow to train) with the following optional keys:

                        Fixed Weights Dictionary Keys (in case of :class:`~psychrnn.backend.models.basic.Basic` and :class:`~psychrnn.backend.models.basic.BasicScan` implementations)
                            * **W_in** (*ndarray(dtype=bool, shape=(:attr:`N_rec`. :attr:`N_in` *)), optional*) -- True for input weights that should be fixed during training.
                            * **W_rec** (*ndarray(dtype=bool, shape=(:attr:`N_rec`, :attr:`N_rec` *)), optional*) -- True for recurrent weights that should be fixed during training.
                            * **W_out** (*ndarray(dtype=bool, shape=(:attr:`N_out`, :attr:`N_rec` *)), optional*) -- True for output weights that should be fixed during training.

                        :Note:
                            In general, any key in the dictionary output by :func:`get_weights` can have a key in the fixed_weights matrix, however fixed_weights will only meaningfully apply to trainable matrices.

                    * **performance_cutoff** (*float*) -- If :data:`performance_measure` is not ``None``, training stops as soon as performance_measure surpases the performance_cutoff. Default: None.
                    * **performance_measure** (*function*) -- Function to calculate the performance of the network using custom criteria. Default: None.

                        :Arguments:
                            * **trial_batch** (*ndarray(dtype=float, shape =(*:attr:`N_batch`, :attr:`N_steps`, :attr:`N_out` *))*): Task stimuli for :attr:`N_batch` trials.
                            * **trial_y** (*ndarray(dtype=float, shape =(*:attr:`N_batch`, :attr:`N_steps`, :attr:`N_out` *))*): Target output for the network on :attr:`N_batch` trials given the :data:`trial_batch`.
                            * **output_mask** (*ndarray(dtype=bool, shape =(*:attr:`N_batch`, :attr:`N_steps`, :attr:`N_out` *))*): Output mask for :attr:`N_batch` trials. True when the network should aim to match the target output, False when the target output can be ignored.
                            * **output** (*ndarray(dtype=bool, shape =(*:attr:`N_batch`, :attr:`N_steps`, :attr:`N_out` *))*): Output to compute the accuracy of. ``output`` as returned by :func:`psychrnn.backend.rnn.RNN.test`.
                            * **epoch** (*int*): Current training epoch (e.g. perhaps the performance_measure is calculated differently early on vs late in training)
                            * **losses** (*list of float*): List of losses from the beginning of training until the current epoch.
                            * **verbosity** (*bool*): Passed in from :data:`train_params`.

                        :Returns:
                            *float* 

                            Performance, greater when the performance is better.
        Returns:
            tuple:
            * **losses** (*list of float*) -- List of losses, computed every :data:`loss_epoch` epochs during training.
            * **training_time** (*float*) -- Time spent training.
            * **initialization_time** (*float*) -- Time spent initializing the network and preparing to train.

        """
        if not self.is_built:
            self.build()

        t0 = time()
        # --------------------------------------------------
        # Extract params
        # --------------------------------------------------
        learning_rate = train_params.get('learning_rate', .001)
        training_iters = train_params.get('training_iters', 50000)
        loss_epoch = train_params.get('loss_epoch', 10)
        verbosity = train_params.get('verbosity', True)
        save_weights_path = train_params.get('save_weights_path', None)
        save_training_weights_epoch = train_params.get('save_training_weights_epoch', 100)
        training_weights_path = train_params.get('training_weights_path', None)
        curriculum = train_params.get('curriculum', None)
        optimizer = train_params.get('optimizer',
                                     tf.compat.v1.train.AdamOptimizer(learning_rate=learning_rate))
        clip_grads = train_params.get('clip_grads', True)
        fixed_weights = train_params.get('fixed_weights', None) # array of zeroes and ones. One indicates to pin and not train that weight.
        performance_cutoff = train_params.get('performance_cutoff', None)
        performance_measure = train_params.get('performance_measure', None)

        if (performance_cutoff is not None and performance_measure is None) or (performance_cutoff is None and performance_measure is not None):
                raise UserWarning("training will not be cutoff based on performance. Make sure both performance_measure and performance_cutoff are defined")

        if curriculum is not None:
            trial_batch_generator = curriculum.get_generator_function()

        if not isgenerator(trial_batch_generator):
            trial_batch_generator = trial_batch_generator.batch_generator()

        # --------------------------------------------------
        # Make weights folder if it doesn't already exist.
        # --------------------------------------------------
        if save_weights_path != None:
            if path.dirname(save_weights_path) != "" and not path.exists(path.dirname(save_weights_path)):
                makedirs(path.dirname(save_weights_path))

        # --------------------------------------------------
        # Make train weights folder if it doesn't already exist.
        # --------------------------------------------------
        if training_weights_path != None:
            if path.dirname(training_weights_path) != "" and not path.exists(path.dirname(training_weights_path)):
                makedirs(path.dirname(_weights_path))

        # --------------------------------------------------
        # Compute gradients
        # --------------------------------------------------
        grads = optimizer.compute_gradients(self.reg_loss)

        # --------------------------------------------------
        # Fixed Weights
        # --------------------------------------------------
        if fixed_weights is not None:
            for i in range(len(grads)):
                (grad, var) = grads[i]
                name = var.name[len(self.name)+1:-2]
                if name in fixed_weights.keys():
                    grad = tf.multiply(grad, (1-fixed_weights[name]))
                    grads[i] = (grad, var)


        # --------------------------------------------------
        # Clip gradients
        # --------------------------------------------------
        if clip_grads:
            grads = [(tf.clip_by_norm(grad, 1.0), var)
                     if grad is not None else (grad, var)
                     for grad, var in grads]

        # --------------------------------------------------
        # Call the optimizer and initialize variables
        # --------------------------------------------------
        optimize = optimizer.apply_gradients(grads)
        self.sess.run(tf.compat.v1.global_variables_initializer())
        self.is_initialized = True

        # --------------------------------------------------
        # Record training time for performance benchmarks
        # --------------------------------------------------
        t1 = time()

        # --------------------------------------------------
        # Training loop
        # --------------------------------------------------
        epoch = 1
        batch_size = next(trial_batch_generator)[0].shape[0]
        losses = []
        if performance_cutoff is not None:
            performance = performance_cutoff - 1

        while epoch * batch_size < training_iters and (performance_cutoff is None or performance < performance_cutoff):
            batch_x, batch_y, output_mask, _ = next(trial_batch_generator)
            self.sess.run(optimize, feed_dict={self.x: batch_x, self.y: batch_y, self.output_mask: output_mask})
            # --------------------------------------------------
            # Output batch loss
            # --------------------------------------------------
            if epoch % loss_epoch == 0:
                reg_loss = self.sess.run(self.reg_loss,
                                feed_dict={self.x: batch_x, self.y: batch_y, self.output_mask: output_mask})
                losses.append(reg_loss)
                if verbosity:
                    print("Iter " + str(epoch * batch_size) + ", Minibatch Loss= " + \
                          "{:.6f}".format(reg_loss))

            # --------------------------------------------------
            # Allow for curriculum learning
            # --------------------------------------------------
            if curriculum is not None and epoch % curriculum.metric_epoch == 0:
                trial_batch, trial_y, output_mask, _ = next(trial_batch_generator)
                output, _ = self.test(trial_batch)
                if curriculum.metric_test(trial_batch, trial_y, output_mask, output, epoch, losses, verbosity):
                    if curriculum.stop_training:
                        break
                    trial_batch_generator = curriculum.get_generator_function()

            # --------------------------------------------------
            # Save intermediary weights
            # --------------------------------------------------
            if epoch % save_training_weights_epoch == 0:
                if training_weights_path is not None:
                    self.save(training_weights_path + str(epoch))
                    if verbosity:
                        print("Training weights saved in file: %s" % training_weights_path + str(epoch))
            
            # ---------------------------------------------------
            # Update performance value if necessary
            # ---------------------------------------------------
            if performance_measure is not None:
                trial_batch, trial_y, output_mask, _ = next(trial_batch_generator)
                output, _ = self.test(trial_batch)
                performance = performance_measure(trial_batch, trial_y, output_mask, output, epoch, losses, verbosity)
                if verbosity:
                    print("performance: " + str(performance))
            epoch += 1

        t2 = time()
        if verbosity:
            print("Optimization finished!")

        # --------------------------------------------------
        # Save final weights
        # --------------------------------------------------
        if save_weights_path is not None:
            self.save(save_weights_path)
            if verbosity:
                print("Model saved in file: %s" % save_weights_path)

        # --------------------------------------------------
        # Return losses, training time, initialization time
        # --------------------------------------------------
        return losses, (t2 - t1), (t1 - t0)


[docs]    def train_curric(self, train_params):
        """Wrapper function for training with curriculum to streamline curriculum learning.

        Arguments: 
            train_params (dict, optional): See :func:`train` for details.

        Returns:
            tuple: See :func:`train` for details.
        """
        # --------------------------------------------------
        # Wrapper function for training with curriculum
        # to streamline curriculum learning
        # --------------------------------------------------

        curriculum = train_params.get('curriculum', None)
        if curriculum is None:
            raise UserWarning("train_curric requires a curriculum. Please pass in a curriculum or use train instead.")
        
        losses, training_time, initialization_time = self.train(curriculum.get_generator_function(), train_params)

        return losses, training_time, initialization_time


[docs]    def test(self, trial_batch):
        """ Test the network on a certain task input.

        Arguments:
            trial_batch ((*ndarray(dtype=float, shape =(*:attr:`N_batch`, :attr:`N_steps`, :attr:`N_out` *))*): Task stimulus to run the network on. Stimulus from :func:`psychrnn.tasks.task.Task.get_trial_batch`, or from next(:func:`psychrnn.tasks.task.Task.batch_generator` ).
        
        Returns:
            tuple:
            * **outputs** (*ndarray(dtype=float, shape =(*:attr:`N_batch`, :attr:`N_steps`, :attr:`N_out` *))*) -- Output time series of the network for each trial in the batch.
            * **states** (*ndarray(dtype=float, shape =(*:attr:`N_batch`, :attr:`N_steps`, :attr:`N_rec` *))*) -- Activity of recurrent units during each trial.
        """
        if not self.is_built:
            self.build()

        if not self.is_initialized:
            self.sess.run(tf.compat.v1.global_variables_initializer())
            self.is_initialized = True

        # --------------------------------------------------
        # Run the forward pass on trial_batch
        # --------------------------------------------------
        outputs, states = self.sess.run([self.predictions, self.states],
                                        feed_dict={self.x: trial_batch})

        return outputs, states
```

---

© Copyright 2020, Authors redacted for double-blind review

Built with Sphinx using a theme provided by Read the Docs.
