# Supplementary material for: PsychRNN: An Accessible and Flexible Python Package for Training Recurrent Neural Network Models on Cognitive Tasks
Source: eNeuro. 2021 Jan 5;8(1):ENEURO.0427-20.2020. doi: 10.1523/ENEURO.0427-20.2020 (PMC7814477; doi:10.1523/ENEURO.0427-20.2020)
Supplement: Extended Data 1 — PsychRNN package and documentation. Download Extended Data 1, ZIP file. [file enu-eN-OTM-0427-20-s06.zip › PsychRNN Extended Data 1/docs/_build/html/_modules/psychrnn/backend/simulation.html]

  


psychrnn.backend.simulation — PsychRNN 1.0.0-alpha documentation


PsychRNN

1.0

Contents:

- Installation Guide
- API Documentation
- Getting Started

PsychRNN

- Docs »
- Module code »
- psychrnn.backend.simulation

---

# Source code for psychrnn.backend.simulation

```
from __future__ import division

import numpy as np

from abc import ABCMeta, abstractmethod

# abstract class python 2 & 3 compatible
ABC = ABCMeta('ABC', (object,), {})

[docs]def relu(x):
    """NumPy implementation of `tf.nn.relu <https://www.tensorflow.org/api_docs/python/tf/nn/relu>`_

    Arguments:
        x (ndarray): array for which relu is computed.

    Returns:
        ndarray: np.maximum(x,0)
    """
    return np.maximum(x, 0)


[docs]def sigmoid(x):
    """NumPy implementation of `tf.nn.sigmoid <https://www.tensorflow.org/api_docs/python/tf/math/sigmoid>`_

    Arguments:
        x (ndarray): array for which sigmoid is computed.

    Returns:
        ndarray: 1/(1 + np.exp(-x))
    """
    return 1/(1 + np.exp(-x))


[docs]class Simulator(ABC):
    """The simulator class.

    Note:
        The base Simulator class is not itself a functioning Simulator. 
        run_trials and rnn_step must be implemented to define a functioning Simulator

    Args:
       rnn_model (:class:`psychrnn.backend.rnn.RNN` object, optional): Uses the :class:`psychrnn.backend.rnn.RNN` object to set :attr:`alpha` and :attr:`rec_noise`. Also used to initialize weights if :data:`weights` and :data:`weights_path` are not passed in. Default: None.
       weights_path (str, optional): Where to load weights from. Take precedence over rnn_model weights. Default: :data:`rnn_model.get_weights() <rnn_model>`. np.load(:data:`weights_path`) should return something of the form :data:`weights`.
       transfer_function (function, optonal): Function that takes an ndarray as input and outputs an ndarray of the same shape with the transfer / activation function applied. NumPy implementation of a TensorFlow transfer function. Default: :func:`relu`.
       weights (dict, optional): Takes precedence over both weights_path and rnn_model. Default: np.load(:data:`weights_path`). Dictionary containing the following keys:

        :Dictionary Keys:
            * **init_state** (*ndarray(dtype=float, shape=(1,* :attr:`N_rec` *))*) -- Initial state of the network's recurrent units.
            * **W_in** (*ndarray(dtype=float, shape=(*:attr:`N_rec`. :attr:`N_in` *))*) -- Input weights.
            * **W_rec** (*ndarray(dtype=float, shape=(*:attr:`N_rec`, :attr:`N_rec` *))*) -- Recurrent weights.
            * **W_out** (*ndarray(dtype=float, shape=(*:attr:`N_out`, :attr:`N_rec` *))*) -- Output weights.
            * **b_rec** (*ndarray(dtype=float, shape=(*:attr:`N_rec`, *))*) -- Recurrent bias.
            * **b_out** (*ndarray(dtype=float, shape=(*:attr:`N_out`, *))*) -- Output bias.

       params (dict, optional):
        :Dictionary Keys:
            * **rec_noise** (*float, optional*) -- Amount of recurrent noise to add to the network. Default: 0
            * **alpha** (*float, optional*) -- The number of unit time constants per simulation timestep. Defaut: (1.0* dt) / tau
            * **dt** (*float, optional*) -- The simulation timestep. Used to calculate alpha if alpha is not passed in. Required if alpha is not in params and rnn_model is None.
            * **tau** (*float*) -- The intrinsic time constant of neural state decay. Used to calculate alpha if alpha is not passed in. Required if alpha is not in params and rnn_model is None.

    """
    def __init__(self,  rnn_model = None, params = None, weights_path=None, weights=None, transfer_function = relu):

        # ----------------------------------
        # Extract params
        # ----------------------------------
        self.transfer_function = transfer_function

        if rnn_model is not None:
            self.alpha = rnn_model.alpha
            self.rec_noise = rnn_model.rec_noise
            if rnn_model.transfer_function.__name__ != self.transfer_function.__name__:
                raise UserWarning("The rnn_model transfer function is " + str(rnn_model.transfer_function) + " and the current transfer function is " + str(self.transfer_function) + ". You should make sure these functions do the same thing -- their names do not match.")
            if params is not None:
                raise UserWarning("params was passed in but will not be used. rnn_model takes precedence.")
        else:
            self.rec_noise = params.get('rec_noise', 0)
            if params.get('alpha') is not None:
                self.alpha = params['alpha']
            else:
                dt = params['dt']
                tau = params['tau']
                self.alpha = params.get('alpha',(1.0* dt) / tau)


        # ----------------------------------
        # Initialize weights
        # ----------------------------------
        self.weights = weights
        if weights is not None:
            if weights_path is not None or rnn_model is not None:
                raise UserWarning("Weights and either rnn_model or weights_path were passed in. Weights from rnn_model and weights_path will be ignored.")
        elif weights_path is not None:
            if rnn_model is not None:
                raise UserWarning("rnn_model and weights_path were both passed in. Weights from rnn_model will be ignored.")
            self.weights = np.load(weights_path)
        elif rnn_model is not None:
            self.weights = rnn_model.get_weights()
        else:
            raise UserWarning("Either weights, rnn_model, or weights_path must be passed in.")

        self.W_in = self.weights['W_in']
        self.W_rec = self.weights['W_rec']
        self.W_out = self.weights['W_out']

        self.b_rec = self.weights['b_rec']
        self.b_out = self.weights['b_out']

        self.init_state = self.weights['init_state']

    # ----------------------------------------------
    # t_connectivity allows for ablation experiments
    # ----------------------------------------------

[docs]    @abstractmethod
    def rnn_step(self, state, rnn_in, t_connectivity):
        """Given input and previous state, outputs the next state and output of the network.

        Note:
            This is an abstract function that must be defined in a child class.

        Arguments:
            state (ndarray(dtype=float, shape=(:attr:`N_batch` , :attr:`N_rec`))): State of network at previous time point.
            rnn_in (ndarray(dtype=float, shape=(:attr:`N_batch` , :attr:`N_in`))): State of network at previous time point.
            t_connectivity (ndarray(dtype=float, shape=(:attr:`N_rec` , :attr:`N_rec`))): Matrix for ablating / perturbing W_rec.

        Returns:
            tuple:
            * **new_output** (*ndarray(dtype=float, shape=(*:attr:`N_batch`, :attr:`N_out` *))*) -- Output of the network at a given timepoint for each trial in the batch.
            * **new_state** (*ndarray(dtype=float, shape=(*:attr:`N_batch`, :attr:`N_rec` *))*) -- New state of the network for each trial in the batch.

        """
        pass


[docs]    @abstractmethod
    def run_trials(self, trial_input, t_connectivity=None):
        """Test the network on a certain task input, optionally including ablation terms.

        A NumPy implementation of :func:`~psychrnn.backend.rnn.RNN.test` with additional options for ablation.

        N_batch here is flexible and will be inferred from trial_input.

        Arguments:
            trial_batch ((*ndarray(dtype=float, shape =(*:attr:`N_batch`, :attr:`N_steps`, :attr:`N_out` *))*): Task stimulus to run the network on. Stimulus from :func:`psychrnn.tasks.task.Task.get_trial_batch`, or from next(:func:`psychrnn.tasks.task.Task.batch_generator` ). If you want the network to run autonomously, without input, set input to an array of zeroes, N_steps will still indicate how long to run the network.
            t_connectivity ((*ndarray(dtype=float, shape =(*:attr:`N_steps`, :attr:`N_rec`, :attr:`N_rec` *))*): Matrix for ablating / perturbing W_rec. Passed step by step to rnn_step.

        Returns:
            tuple:
            * **outputs** (*ndarray(dtype=float, shape =(*:attr:`N_batch`, :attr:`N_steps`, :attr:`N_out` *))*) -- Output time series of the network for each trial in the batch.
            * **states** (*ndarray(dtype=float, shape =(*:attr:`N_batch`, :attr:`N_steps`, :attr:`N_rec` *))*) -- Activity of recurrent units during each trial.
        """
        pass


[docs]class BasicSimulator(Simulator):
    """:class:`Simulator` implementation for :class:`psychrnn.backend.models.basic.Basic` and for :class:`psychrnn.backend.models.basic.BasicScan`.

    See :class:`Simulator` for arguments.
    """

[docs]    def rnn_step(self, state, rnn_in, t_connectivity):
        """Given input and previous state, outputs the next state and output of the network as a NumPy implementation of :class:`psychrnn.backend.models.basic.Basic.recurrent_timestep` and of :class:`psychrnn.backend.models.basic.Basic.output_timestep`.

        Additionally takes in :data:`t_connectivity`. If :data:`t_connectivity` is all ones, :func:`rnn_step`'s output will match that of :class:`psychrnn.backend.models.basic.Basic.recurrent_timestep` and :class:`psychrnn.backend.models.basic.Basic.output_timestep`. Otherwise :data:`W_rec` is multiplied by :data:`t_connectivity` elementwise, ablating / perturbing the recurrent connectivity.

        Arguments:
            state (ndarray(dtype=float, shape=(:attr:`N_batch` , :attr:`N_rec`))): State of network at previous time point.
            rnn_in (ndarray(dtype=float, shape=(:attr:`N_batch` , :attr:`N_in`))): State of network at previous time point.
            t_connectivity (ndarray(dtype=float, shape=(:attr:`N_rec` , :attr:`N_rec`))): Matrix for ablating / perturbing W_rec.

        Returns:
            tuple:
            * **new_output** (*ndarray(dtype=float, shape=(*:attr:`N_batch`, :attr:`N_out` *))*) -- Output of the network at a given timepoint for each trial in the batch.
            * **new_state** (*ndarray(dtype=float, shape=(*:attr:`N_batch`, :attr:`N_rec` *))*) -- New state of the network for each trial in the batch.

        """

        new_state = ((1-self.alpha) * state) \
                    + self.alpha * (
                        np.matmul(
                            self.transfer_function(state),
                            np.transpose(self.W_rec * t_connectivity))
                        + np.matmul(rnn_in, np.transpose(self.W_in))
                        + self.b_rec)\
                    + np.sqrt(2.0 * self.alpha * self.rec_noise * self.rec_noise) * \
                      np.random.normal(loc=0.0, scale=1.0, size=state.shape)

        new_output = np.matmul(
                        self.transfer_function(new_state),
                        np.transpose(self.W_out)) + self.b_out

        return new_output, new_state


[docs]    def run_trials(self, trial_input, t_connectivity=None):
        """Test the network on a certain task input, optionally including ablation terms.

        A NumPy implementation of :func:`~psychrnn.backend.rnn.RNN.test` with additional options for ablation.

        N_batch here is flexible and will be inferred from trial_input.

        Repeatedly calls :func:`rnn_step` to build output and states over the entire timecourse of the :data:`trial_batch`

        Arguments:
            trial_batch ((*ndarray(dtype=float, shape =(*:attr:`N_batch`, :attr:`N_steps`, :attr:`N_out` *))*): Task stimulus to run the network on. Stimulus from :func:`psychrnn.tasks.task.Task.get_trial_batch`, or from next(:func:`psychrnn.tasks.task.Task.batch_generator` ).
                   To run the network autonomously without input, set input to an array of zeroes. N_steps will still indicate for how many steps to run the network.
            t_connectivity ((*ndarray(dtype=float, shape =(*:attr:`N_steps`, :attr:`N_rec`, :attr:`N_rec` *))*): Matrix for ablating / perturbing W_rec. Passed step by step to :func:`rnn_step`.

        Returns:
            tuple:
            * **outputs** (*ndarray(dtype=float, shape =(*:attr:`N_batch`, :attr:`N_steps`, :attr:`N_out` *))*) -- Output time series of the network for each trial in the batch.
            * **states** (*ndarray(dtype=float, shape =(*:attr:`N_batch`, :attr:`N_steps`, :attr:`N_rec` *))*) -- Activity of recurrent units during each trial.
        """

        batch_size = trial_input.shape[0]
        rnn_inputs = np.squeeze(np.split(trial_input, trial_input.shape[1], axis=1))
        state = np.expand_dims(self.init_state[0, :], 0)
        state = np.repeat(state, batch_size, 0)
        rnn_outputs = []
        rnn_states = []
        for i, rnn_input in enumerate(rnn_inputs):

            if t_connectivity is not None:
                output, state = self.rnn_step(state, rnn_input, t_connectivity[i])
            else:
                output, state = self.rnn_step(state, rnn_input, np.ones_like(self.W_rec))

            rnn_outputs.append(output)
            rnn_states.append(state)

        return np.swapaxes(np.array(rnn_outputs), 0, 1), np.swapaxes(np.array(rnn_states), 0, 1)


[docs]class LSTMSimulator(Simulator):
    """:class:`Simulator` implementation for :class:`psychrnn.backend.models.lstm.LSTM` and for :class:`psychrnn.backend.models.lstm.LSTM`.

    See :class:`Simulator` for arguments.

    The contents of weights / np.load(weights_path) must now include the following additional keys:

    :Dictionary Keys:
        * **init_hidden** (*ndarray(dtype=float, shape=(*:attr:`N_batch` , :attr:`N_rec` *))*) -- Initial state of the cell state.
        * **init_hidden** (*ndarray(dtype=float, shape=(*:attr:`N_batch` , :attr:`N_rec` *))*) -- Initial state of the hidden state.
        * **W_f** (*ndarray(dtype=float, shape=(*:attr:`N_rec` + :attr:`N_in`, :attr:`N_rec` *))*) -- f term weights
        * **W_i** (*ndarray(dtype=float, shape=(*:attr:`N_rec` + :attr:`N_in`, :attr:`N_rec` *))*) -- i term weights
        * **W_c** (*ndarray(dtype=float, shape=(*:attr:`N_rec` + :attr:`N_in`, :attr:`N_rec` *))*) -- c term weights
        * **W_o** (*ndarray(dtype=float, shape=(*:attr:`N_rec` + :attr:`N_in`, :attr:`N_rec` *))*) -- o term weights
        * **b_f** (*ndarray(dtype=float, shape=(*:attr:`N_rec`, *))*) -- f term bias.
        * **b_i** (*ndarray(dtype=float, shape=(*:attr:`N_rec`, *))*) -- i term bias.
        * **b_c** (*ndarray(dtype=float, shape=(*:attr:`N_rec`, *))*) -- c term bias.
        * **b_o** (*ndarray(dtype=float, shape=(*:attr:`N_rec`, *))*) -- o term bias.
    """

    def __init__(self,  rnn_model = None, params = None, weights_path=None, weights=None):

        super(LSTMSimulator, self).__init__(rnn_model=rnn_model, params=params, weights_path=weights_path, weights=weights)

        self.init_hidden = self.weights['init_hidden']
        self.init_cell = self.weights['init_cell']

        self.W_f = self.weights['W_f']
        self.W_i = self.weights['W_i']
        self.W_c = self.weights['W_c']
        self.W_o = self.weights['W_o']

        self.b_f = self.weights['b_f']
        self.b_i = self.weights['b_i']
        self.b_c = self.weights['b_c']
        self.b_o = self.weights['b_o']

[docs]    def rnn_step(self, hidden, cell, rnn_in):
        """Given input and previous state, outputs the next state and output of the network as a NumPy implementation of :class:`psychrnn.backend.models.lstm.LSTM.recurrent_timestep` and of :class:`psychrnn.backend.models.lstm.LSTM.output_timestep`.

        Arguments:
            hidden (ndarray(dtype=float, shape=(:attr:`N_batch` , :attr:`N_rec` ))): Hidden units state of network at previous time point.
            cell (ndarray(dtype=float, shape=(:attr:`N_batch` , :attr:`N_rec` ))): Cell state of the network at previous time point.
            rnn_in (ndarray(dtype=float, shape=(:attr:`N_batch` , :attr:`N_in`))): State of network at previous time point.

        Returns:
            tuple:
            * **new_output** (*ndarray(dtype=float, shape=(*:attr:`N_batch`, :attr:`N_out` *))*) -- Output of the network at a given timepoint for each trial in the batch.
            * **new_hidden** (*ndarray(dtype=float, shape=(* :attr:`N_batch` , :attr:`N_rec` *))*) -- New hidden unit state of the network.
            * **new_cell** (*ndarray(dtype=float, shape=(* :attr:`N_batch` , :attr:`N_rec` *))*) -- New cell state of the network.

        """

        f = sigmoid(np.matmul(np.concatenate([hidden, rnn_in], 1), self.W_f)
                               + self.b_f)

        i = sigmoid(np.matmul(np.concatenate([hidden, rnn_in], 1), self.W_i)
                               + self.b_i)

        c = np.tanh(np.matmul(np.concatenate([hidden, rnn_in], 1), self.W_c)
                               + self.b_c)

        o = sigmoid(np.matmul(np.concatenate([hidden, rnn_in], 1), self.W_o)
                          + self.b_o)

        new_cell = f * cell + i * c

        new_hidden = o * sigmoid(new_cell)

        new_output = np.matmul(new_hidden, np.transpose(self.W_out)) + self.b_out

        return new_output, new_hidden, new_cell


[docs]    def run_trials(self, trial_input):
        """Test the network on a certain task input, optionally including ablation terms.

        A NumPy implementation of :func:`~psychrnn.backend.rnn.RNN.test` with additional options for ablation.

        N_batch here is flexible and will be inferred from trial_input.

        Repeatedly calls :func:`rnn_step` to build output and states over the entire timecourse of the :data:`trial_batch`

        Arguments:
            trial_batch ((*ndarray(dtype=float, shape =(*:attr:`N_batch`, :attr:`N_steps`, :attr:`N_out` *))*): Task stimulus to run the network on. Stimulus from :func:`psychrnn.tasks.task.Task.get_trial_batch`, or from next(:func:`psychrnn.tasks.task.Task.batch_generator` ).
                   To run the network autonomously without input, set input to an array of zeroes. N_steps will still indicate for how many steps to run the network.

        Returns:
            tuple:
            * **outputs** (*ndarray(dtype=float, shape =(*:attr:`N_batch`, :attr:`N_steps`, :attr:`N_out` *))*) -- Output time series of the network for each trial in the batch.
            * **states** (*ndarray(dtype=float, shape =(*:attr:`N_batch`, :attr:`N_steps`, :attr:`N_rec` *))*) -- Activity of recurrent units during each trial.
        """

        batch_size = trial_input.shape[0]
        rnn_inputs = np.squeeze(np.split(trial_input, trial_input.shape[1], axis=1))
        cell = np.expand_dims(self.init_cell[0, :], 0)
        cell = np.repeat(cell, batch_size, 0)
        hidden = np.expand_dims(self.init_hidden[0, :], 0)
        hidden = np.repeat(hidden, batch_size, 0)
        rnn_outputs = []
        rnn_states = []
        for i, rnn_input in enumerate(rnn_inputs):

            output, hidden, cell = self.rnn_step(hidden, cell, rnn_input)

            rnn_outputs.append(output)
            rnn_states.append(hidden)

        return np.swapaxes(np.array(rnn_outputs), 0, 1), np.swapaxes(np.array(rnn_states), 0, 1)
```

---

© Copyright 2020, Authors redacted for double-blind review

Built with Sphinx using a theme provided by Read the Docs.
