# Supplementary material for: PsychRNN: An Accessible and Flexible Python Package for Training Recurrent Neural Network Models on Cognitive Tasks
Source: eNeuro. 2021 Jan 5;8(1):ENEURO.0427-20.2020. doi: 10.1523/ENEURO.0427-20.2020 (PMC7814477; doi:10.1523/ENEURO.0427-20.2020)
Supplement: Extended Data 1 — PsychRNN package and documentation. Download Extended Data 1, ZIP file. [file enu-eN-OTM-0427-20-s06.zip › PsychRNN Extended Data 1/docs/_build/html/_modules/psychrnn/backend/curriculum.html]

  


psychrnn.backend.curriculum — PsychRNN 1.0.0-alpha documentation


PsychRNN

1.0

Contents:

- Installation Guide
- API Documentation
- Getting Started

PsychRNN

- Docs »
- Module code »
- psychrnn.backend.curriculum

---

# Source code for psychrnn.backend.curriculum

```
from __future__ import division
from __future__ import print_function

from os import makedirs, path
import numpy as np

[docs]def default_metric(curriculum_params, input_data, correct_output, output_mask, output, epoch, losses, verbosity):
	""" Default metric to use to evaluate performance when using Curriculum learning.

	Advance is true if accuracy >= threshold, False otherwise.

	Arguments:
		curriculum_params (dict): Dictionary of the :class:`Curriculum` object parameters, containing the following keys:

			:Dictionary Keys:
				* **stop_training** (*bool*) -- True if the network has finished training and completed all stages.
				* **stage** (*int*) -- Current training stage (initial stage is 0).
				* **metric_values** (*list of [float, int]*)  -- List of metric values and the stage at which each metric value was computed.
				* **tasks** (*list of :class:`psychrnn.tasks.task.Task` objects*) -- List of tasks in the curriculum.
				* **metric** (*function*) -- What metric function to use. :func:`default_metric` is an example of one in terms of inputs and outputs taken.
				* **accuracies** (*list of functions with the signature of* :func:`psychrnn.tasks.task.Task.accuracy_function`) -- Accuracy function to use at each stage.
				* **thresholds** (*list of float*) -- Thresholds for each stage that accuracy must reach to move to the next stage.
				* **metric_epoch** (*int*) -- Calculate the metric / test if advance to the next stage every metric_epoch training epochs.
				* **output_file** (*str*) -- Optional path for where to save out metric value and stage.

		input_data (ndarray(dtype=float, shape =(:attr:`N_batch`, :attr:`N_steps`, :attr:`N_out` ))): Task inputs.
		correct_output (ndarray(dtype=float, shape = (:attr:`N_batch`, :attr:`N_steps`, :attr:`N_out`))): Correct (target) task output given input_data.
		output_mask (ndarray(dtype=float, shape = (:attr:`N_batch`, :attr:`N_steps`, :attr:`N_out`))): Output mask for the task. True when the network should aim to match the target output, False when the target output can be ignored.
		output (ndarray(dtype=float, shape = (:attr:`N_batch`, :attr:`N_steps`, :attr:`N_out`))): The network's output given input_data.
		epoch (*int*): The epoch number in training.
		losses (*list of float*): List of losses, computed during training.
		verbosity (*bool*): Whether to print information as training progresses. If True, prints accuracy every time it is computed.

	Returns:
		tuple:

		* **advance** (*bool*) -- True if the accuracy is >= the threshold for the current stage. False otherwise.
		* **metric_value** (*float*) -- Value of the computed accuracy.

	"""
	accuracy = curriculum_params['accuracies'][curriculum_params['stage']](correct_output,output, output_mask)
	threshold = curriculum_params['thresholds'][curriculum_params['stage']]
	if verbosity:
		print("Accuracy: " + str(accuracy))
	return accuracy>=threshold, accuracy


[docs]class Curriculum(object):
	""" Curriculum object.

	Allows training on a sequence of tasks when Curriculum is passed into :func:`~psychrnn.backend.rnn.RNN.train`.

	Arguments:
		tasks (list of :class:`~psychrnn.tasks.task.Task` objects):  List of tasks  to use in the curriculum.
		metric (function, optional): Function for calculating whether the stage advances and what the metric value is at each metric_epoch. Default: :func:`default_metric`.

			:Arguments:
				* **curriculum_params** (*dict*) -- Dictionary of the :class:`Curriculum` object parameters, containing the following keys:

				:Dictionary Keys:
					* **stop_training** (*bool*) -- True if the network has finished training and completed all stages.
					* **stage** (*int*) -- Current training stage (initial stage is 0).
					* **metric_values** (*list of [float, int]*)  -- List of metric values and the stage at which each metric value was computed.
					* **tasks** (*list of :class:`psychrnn.tasks.task.Task` objects*) -- List of tasks in the curriculum.
					* **metric** (*function*) -- What metric function to use. :func:`default_metric` is an example of one in terms of inputs and outputs taken.
					* **accuracies** (*list of functions*) -- Accuracy function to use at each stage.
					* **thresholds** (*list of float*) -- Thresholds for each stage that accuracy must reach to move to the next stage.
					* **metric_epoch** (*int*) -- Calculate the metric and test if the model should advance to the next stage every :data:`metric_epoch` training epochs.
					* **output_file** (*str*) -- Optional path for saving out themetric value and stage. If the .npz filename extension is not included, it will be appended.

				* **input_data** (*ndarray(dtype=float, shape =(*:attr:`N_batch`, :attr:`N_steps`, :attr:`N_out` *))*) -- Task inputs.
				* **correct_output** (*ndarray(dtype=float, shape = (*:attr:`N_batch`, :attr:`N_steps`, :attr:`N_out` *))*) -- Correct (target) task output given input_data.
				* **output_mask** (*ndarray(dtype=float, shape = (*:attr:`N_batch`, :attr:`N_steps`, :attr:`N_out` *))*) -- Output mask for the task. True when the network should aim to match the target output, False when the target output can be ignored.
				* **output** (*ndarray(dtype=float, shape = (*:attr:`N_batch`, :attr:`N_steps`, :attr:`N_out` *))*) -- The network's output given input_data.
				* **epoch** (*int*) -- The epoch number in training.
				* **losses** (*list of float*) -- List of losses, computed during training.
				* **verbosity** (*bool*) -- Whether to print information as training progresses.

			:Returns:
				*tuple*

				* **advance** (*bool*) -- True if the the stage should be advanced. False otherwise.
				* **metric_value** (*float*) -- Value of the computed metric.

		accuracies (list of functions, optional): Optional list of functions to use to calculate network performance for the purposes of advancing tasks. Used by :func:`default_metric` to compute accuracy. Default: ``[tasks[i].accuracy_function for i in range(len(tasks))]``.
		thresholds (list of float, optional): Optional list of thresholds. If metric = default_metric, accuracies must reach the threshold for a given stage in order to advance to the next stage. Default: ``[.9 for i in range(len(tasks))]``
		metric_epoch (int): Calculate the metric and test if the model should advance to the next stage every :data:`metric_epoch` training epochs. Default: 10
		output_file (str): Optional path for saving out the metric value and stage. If the .npz filename extension is not included, it will be appended. Default: None.

	"""
	def __init__(self, tasks, **kwargs):
		self.stop_training = False
		self.stage = 0
		self.metric_values = []

		# List of tasks that make up the curriculum
		self.tasks = tasks

		#Optional function with parameters as in default_metric that returns whether to advance stage, and the accuracy / metric value
		self.metric = kwargs.get('metric', default_metric)
		
		#Optional list of accuracy functions to use for each task
		self.accuracies = kwargs.get('accuracies', [tasks[i].accuracy_function for i in range(len(tasks))])
		assert len(self.accuracies)==len(self.tasks)
		
		# Optional list of accuracy cuttoff values to use with each tasks
		self.thresholds = kwargs.get('thresholds', [.9 for i in range(len(tasks))])
		assert len(self.thresholds)==len(self.tasks)
		
		# How often to check metric?
		self.metric_epoch = kwargs.get('metric_epoch', 10)
		
		# Optional path to save out metric value and stage to
		self.output_file = kwargs.get('output_file', None)
		if self.output_file is not None:
			if path.dirname(self.output_file) != "" and not path.exists(path.dirname(self.output_file)):
				makedirs(path.dirname(self.output_file))

[docs]	def metric_test(self, input_data, correct_output, output_mask, test_output, epoch, losses, verbosity = False):
		"""Evaluates whether to advance the stage to the next task or not.

		Arguments:
			input_data (ndarray(dtype=float, shape =(:attr:`N_batch`, :attr:`N_steps`, :attr:`N_out` ))): Task inputs.
			correct_output (ndarray(dtype=float, shape = (:attr:`N_batch`, :attr:`N_steps`, :attr:`N_out`))): Correct (target) task output given input_data.
			output_mask (ndarray(dtype=float, shape = (:attr:`N_batch`, :attr:`N_steps`, :attr:`N_out`))): Output mask for the task. True when the network should aim to match the target output, False when the target output can be ignored.
			test_output (ndarray(dtype=float, shape = (:attr:`N_batch`, :attr:`N_steps`, :attr:`N_out`))): The network's output given input_data.
			epoch (*int*): The epoch number in training.
			losses (*list of float*): List of losses, computed during training.
			verbosity (*bool, optional*): Whether to print information as metric is computed and stages advanced. Default: False

		Returns:
			True if stage advances, False otherwise.
		"""
		advance, metric_value = self.metric(self.__dict__, input_data, correct_output, output_mask, test_output, epoch, losses, verbosity)
		self.metric_values.append([metric_value, self.stage])
		if advance:
			self.stage+=1
			if self.stage == len(self.tasks):
				self.stop_training = True
				if self.output_file is not None:
					np.save(self.output_file, self.metric_values)
					if verbosity:
						print("Metric values saved in file: %s" % self.output_file)
			if verbosity:
				print("Stage " + str(self.stage))
			return True
		return False


[docs]	def get_generator_function(self):
		""" Return the generator function for the current task.

		Returns:
			:func:`psychrnn.tasks.task.Task.batch_generator` function: Task batch generator for the task at the current stage.
		"""
		return self.tasks[self.stage].batch_generator()
```

---

© Copyright 2020, Authors redacted for double-blind review

Built with Sphinx using a theme provided by Read the Docs.
