# Supplementary material for: PsychRNN: An Accessible and Flexible Python Package for Training Recurrent Neural Network Models on Cognitive Tasks
Source: eNeuro. 2021 Jan 5;8(1):ENEURO.0427-20.2020. doi: 10.1523/ENEURO.0427-20.2020 (PMC7814477; doi:10.1523/ENEURO.0427-20.2020)
Supplement: Extended Data 1 — PsychRNN package and documentation. Download Extended Data 1, ZIP file. [file enu-eN-OTM-0427-20-s06.zip › PsychRNN Extended Data 1/docs/_build/html/_modules/psychrnn/backend/loss_functions.html]

  


psychrnn.backend.loss\_functions — PsychRNN 1.0.0-alpha documentation


PsychRNN

1.0

Contents:

- Installation Guide
- API Documentation
- Getting Started

PsychRNN

- Docs »
- Module code »
- psychrnn.backend.loss\_functions

---

# Source code for psychrnn.backend.loss\_functions

```
from __future__ import division

import tensorflow as tf
tf.compat.v1.disable_eager_execution()

[docs]class LossFunction(object):
    """ Set the loss function for the :class:`~psychrnn.backend.rnn.RNN` model.

    Arguments:
        params(dict): Dictionary of parameters including the following keys:

            :Dictionary Keys:
                * **loss_function** (*str*) -- String indicating what loss function to use. If :data:`params["loss_function"]` is not `mean_squared_error` or `binary_cross_entropy`, :data:`params[params["loss_function"]]` defines the custom loss function. Default: "mean_squared_error".
                * **params["loss_function"]** (*function, optional*) -- Defines the custom loss function. Must have the same signature as :func:`mean_squared_error` and :func:`binary_cross_entropy`.

    """

    def __init__(self, params):
        self.type = params.get("loss_function", "mean_squared_error")
        if self.type != "mean_squared_error" and self.type != "binary_cross_entropy":
            self.custom_loss_function = params.get(self.type, None)
            if self.custom_loss_function is None:
                raise UserWarning("Loss type is '" + self.type + "' but '" + self.type + "' is not an entry in params. Did you mean 'mean_square_error' or 'binary_cross_entropy'? If not, you must pass a function in to params as '" + self.type + "'." )

[docs]    def set_model_loss(self, model):
        """ Returns the model loss, calculated as indicated by :attr:`type` (inferred from :data:`params["loss_function"]`.

        ``'mean_squared_error'`` indicates :func:`mean_squared_error`, ``'binary_cross_entropy'`` indicates :func:`binary_cross_entropy`.
        If :attr:`type` is not one of the above options, :attr:`custom_loss_function` is used. The custom loss function would have been passed in to :data:`params` as :data:`params[type]`.

        Args:
            model (:class:`~psychrnn.backend.rnn.RNN` object): Model for which to calculate the regularization.

        Returns:
            tf.Tensor(dtype=float): Model loss.

        """

        loss = 0

        if self.type == "mean_squared_error":
            loss = self.mean_squared_error(model.predictions, model.y, model.output_mask)

        elif self.type == "binary_cross_entropy":
            loss = self.binary_cross_entropy(model.predictions, model.y, model.output_mask)

        else:
            loss = self.custom_loss_function(model.predictions, model.y, model.output_mask)

        return loss


[docs]    def mean_squared_error(self, predictions, y, output_mask):
        """ Mean squared error.

        ``loss = mean(square(output_mask * (predictions - y)))``

        Args:
            predictions (*tf.Tensor(dtype=float, shape =(*:attr:`N_batch`, :attr:`N_steps`, :attr:`N_out` *))*): Network output.
            y (*tf.Tensor(dtype=float, shape =(*?, :attr:`N_steps`, :attr:`N_out` *))*): Target output.
            output_mask (*tf.Tensor(dtype=float, shape =(*?, :attr:`N_steps`, :attr:`N_out` *))*): Output mask for :attr:`N_batch` trials. True when the network should aim to match the target output, False when the target output can be ignored.

        Returns:
            tf.Tensor(dtype=float): Mean squared error.

        """

        return tf.reduce_mean(input_tensor=tf.square(output_mask * (predictions - y)))


[docs]    def binary_cross_entropy(self, predictions, y, output_mask):
        """ Binary cross-entropy.

        Binary label values are assumed to be 0 and 1. 

        ``loss = mean(output_mask * -(y * log(predictions) + (1-y)* log(1-predictions)))``

        Args:
            predictions (*tf.Tensor(dtype=float, shape =(*:attr:`N_batch`, :attr:`N_steps`, :attr:`N_out` *))*): Network output.
            y (*tf.Tensor(dtype=float, shape =(*?, :attr:`N_steps`, :attr:`N_out` *))*): Target output.
            output_mask (*tf.Tensor(dtype=float, shape =(*?, :attr:`N_steps`, :attr:`N_out` *))*): Output mask for :attr:`N_batch` trials. True when the network should aim to match the target output, False when the target output can be ignored.

        Returns:
            tf.Tensor(dtype=float): Binary cross-entropy.

        """

        epsilon = 1e-07 # default epsilon used in TensorFlow
        predictions = tf.clip_by_value(predictions, epsilon, 1. - epsilon)

        return tf.reduce_mean( input_tensor=output_mask *
                               -(y * tf.math.log(predictions + epsilon) + (1 - y) * tf.math.log(1 - predictions + epsilon)))
```

---

© Copyright 2020, Authors redacted for double-blind review

Built with Sphinx using a theme provided by Read the Docs.
