# Supplementary material for: PsychRNN: An Accessible and Flexible Python Package for Training Recurrent Neural Network Models on Cognitive Tasks
Source: eNeuro. 2021 Jan 5;8(1):ENEURO.0427-20.2020. doi: 10.1523/ENEURO.0427-20.2020 (PMC7814477; doi:10.1523/ENEURO.0427-20.2020)
Supplement: Extended Data 1 — PsychRNN package and documentation. Download Extended Data 1, ZIP file. [file enu-eN-OTM-0427-20-s06.zip › PsychRNN Extended Data 1/docs/_build/html/_modules/psychrnn/backend/regularizations.html]

  


psychrnn.backend.regularizations — PsychRNN 1.0.0-alpha documentation


PsychRNN

1.0

Contents:

- Installation Guide
- API Documentation
- Getting Started

PsychRNN

- Docs »
- Module code »
- psychrnn.backend.regularizations

---

# Source code for psychrnn.backend.regularizations

```
from __future__ import division

import tensorflow as tf

tf.compat.v1.disable_eager_execution()

[docs]class Regularizer(object):
    """Regularizer Class

    Class that aggregates all types of regularization used.

    Args:
       params (dict): The regularization parameters containing the following optional keys:

           :Dictionary Keys:
                * **L1_in** (*float, optional*) -- Parameter for weighting the L1 input weights regularization. Default: 0.
                * **L1_rec** (*float, optional*) -- Parameter for weighting the L1 recurrent weights regularization. Default: 0.
                * **L1_out** (*float, optional*) -- Parameter for weighting the L1 output weights regularization. Default: 0.
                * **L2_in** (*float, optional*) -- Parameter for weighting the L2 input weights regularization. Default: 0.
                * **L2_rec** (*float, optional*) -- Parameter for weighting the L2 recurrent weights regularization. Default: 0.
                * **L2_out** (*float, optional*) -- Parameter for weighting the L2 output weights regularization. Default: 0.
                * **L2_firing_rate** (*float, optional*) -- Parameter for weighting the L2 regularization of the relu thresholded states. Default: 0.
                * **custom_regularization** (*function, optional*) -- Custom regularization function. Default: None.

                    Args:
                        * **model** (:class:`~psychrnn.backend.rnn.RNN` *object*) -- Model for which to calculate the regularization.
                        * **params** (*dict*) -- Regularization parameters. All params passed to the :class:`Regularizer` will be passed here.

                    Returns:
                        tf.Tensor(dtype=float): The custom regularization to add when calculating the loss.
    """

    def __init__(self, params):
        # ----------------------------------
        # regularization coefficients
        # ----------------------------------
        self.L1_in = params.get('L1_in', 0)
        self.L1_rec = params.get('L1_rec', 0)
        self.L1_out = params.get('L1_out', 0)

        self.L2_in = params.get('L2_in', 0)
        self.L2_rec = params.get('L2_rec', 0)
        self.L2_out = params.get('L2_out', 0)

        self.L2_firing_rate = params.get('L2_firing_rate', 0)

        self.custom_regularization =  params.get('custom_regularization', None)
        self.params = params

[docs]    def set_model_regularization(self, model):
        """ Given model, calculate the regularization by adding all regualarization terms (scaled with the parameters to be either zero or nonzero).

        The following regularizations are added: :func:`L1_weight_reg`, :func:`L2_weight_reg`, and :func:`L2_firing_rate_reg`.

        Args:
            model (:class:`~psychrnn.backend.rnn.RNN` object): Model for which to calculate the regularization.

        Returns:
            tf.Tensor(dtype=float): The regularization to add when calculating the loss.
        """
        reg = 0

        # ----------------------------------
        # L1 weight regularization
        # ----------------------------------
        reg += self.L1_weight_reg(model)

        # ----------------------------------
        # L2 weight regularization
        # ----------------------------------
        reg += self.L2_weight_reg(model)

        # ----------------------------------
        # L2 firing rate regularization
        # ----------------------------------
        reg += self.L2_firing_rate_reg(model)


        if self.custom_regularization is not None:
            reg += self.custom_regularization(model, self.params)

        return reg


[docs]    def L1_weight_reg(self, model):
        """ L1 regularization

        :math:`regularization = L1\\_in * ||W\\_in||_1 + L1\\_rec * ||W\\_rec||_1 + L1\\_out * ||W\\_out||_1`

        Args:
            model (:class:`~psychrnn.backend.rnn.RNN` object): Model for which to calculate the regularization.

        Returns:
            tf.Tensor(dtype=float): The L1 regularization to add when calculating the loss.
        """

        reg = 0

        reg += self.L1_in * tf.reduce_mean(input_tensor=tf.abs(model.get_effective_W_in()))
        reg += self.L1_rec * tf.reduce_mean(input_tensor=tf.abs(model.get_effective_W_rec()))
        reg += self.L1_out * tf.reduce_mean(input_tensor=tf.abs(model.get_effective_W_out()))

        return reg


[docs]    def L2_weight_reg(self, model):
        """ L2 regularization

        :math:`regularization = L2\\_in * ||W\\_in||_2^2 + L2\\_rec * ||W\\_rec||_2^2 + L2\\_out * ||W\\_out||_2^2`

        Args:
            model (:class:`~psychrnn.backend.rnn.RNN` object): Model for which to calculate the regularization.

        Returns:
            tf.Tensor(dtype=float): The L2 regularization to add when calculating the loss.
        """

        reg = 0

        reg += self.L2_in * tf.reduce_mean(input_tensor=tf.square(tf.abs(model.get_effective_W_in())))
        reg += self.L2_rec * tf.reduce_mean(input_tensor=tf.square(tf.abs(model.get_effective_W_rec())))
        reg += self.L2_out * tf.reduce_mean(input_tensor=tf.square(tf.abs(model.get_effective_W_out())))

        return reg


[docs]    def L2_firing_rate_reg(self, model):
        """ L2 regularization of the firing rate.

        :math:`regularization = L2\\_firing\\_rate * ||relu(states)||_2^2`

        Args:
            model (:class:`~psychrnn.backend.rnn.RNN` object): Model for which to calculate the regularization.

        Returns:
            tf.Tensor(dtype=float): The L2 firing rate regularization to add when calculating the loss.
        """

        reg = self.L2_firing_rate * tf.reduce_mean(input_tensor=tf.square(model.transfer_function(model.states)))

        return reg
```

---

© Copyright 2020, Authors redacted for double-blind review

Built with Sphinx using a theme provided by Read the Docs.
