# Supplementary figures and images for: PsychRNN: An Accessible and Flexible Python Package for Training Recurrent Neural Network Models on Cognitive Tasks
Source: eNeuro. 2021 Jan 5;8(1):ENEURO.0427-20.2020. doi: 10.1523/ENEURO.0427-20.2020 (PMC7814477; doi:10.1523/ENEURO.0427-20.2020)

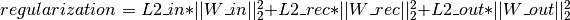

Supplement: Extended Data 1 — PsychRNN package and documentation. Download Extended Data 1, ZIP file. [file enu-eN-OTM-0427-20-s06.zip › PsychRNN Extended Data 1/docs/_build/html/_images/math/6d43b224ffea8e1f929ba71522405d2c6e85906a.png]

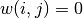

Supplement: Extended Data 1 — PsychRNN package and documentation. Download Extended Data 1, ZIP file. [file enu-eN-OTM-0427-20-s06.zip › PsychRNN Extended Data 1/docs/_build/html/_images/math/0f93e9c3488abbd51931c303d576c7a426e31050.png]

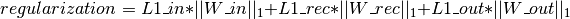

Supplement: Extended Data 1 — PsychRNN package and documentation. Download Extended Data 1, ZIP file. [file enu-eN-OTM-0427-20-s06.zip › PsychRNN Extended Data 1/docs/_build/html/_images/math/a884c4d2d96500d8e8d9518e0507886ab04bc428.png]

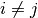

Supplement: Extended Data 1 — PsychRNN package and documentation. Download Extended Data 1, ZIP file. [file enu-eN-OTM-0427-20-s06.zip › PsychRNN Extended Data 1/docs/_build/html/_images/math/756bd1fc8e05d932f03b833058374848f96c41ec.png]

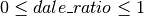

Supplement: Extended Data 1 — PsychRNN package and documentation. Download Extended Data 1, ZIP file. [file enu-eN-OTM-0427-20-s06.zip › PsychRNN Extended Data 1/docs/_build/html/_images/math/6d2bb4ebe4d9f8f0f3230f42936cec6691ae1e8e.png]

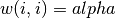

Supplement: Extended Data 1 — PsychRNN package and documentation. Download Extended Data 1, ZIP file. [file enu-eN-OTM-0427-20-s06.zip › PsychRNN Extended Data 1/docs/_build/html/_images/math/69cfc422d42888769b1c7ef6b9d2e1e9f93d7b50.png]

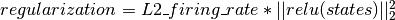

Supplement: Extended Data 1 — PsychRNN package and documentation. Download Extended Data 1, ZIP file. [file enu-eN-OTM-0427-20-s06.zip › PsychRNN Extended Data 1/docs/_build/html/_images/math/af0ecb2f0a68f23e17bd0f5dafab5f7ec0655438.png]
