# Supplementary material for: PsychRNN: An Accessible and Flexible Python Package for Training Recurrent Neural Network Models on Cognitive Tasks
Source: eNeuro. 2021 Jan 5;8(1):ENEURO.0427-20.2020. doi: 10.1523/ENEURO.0427-20.2020 (PMC7814477; doi:10.1523/ENEURO.0427-20.2020)
Supplement: Extended Data 1 — PsychRNN package and documentation. Download Extended Data 1, ZIP file. [file enu-eN-OTM-0427-20-s06.zip › PsychRNN Extended Data 1/docs/_build/html/_modules/psychrnn/backend/initializations.html]

  


psychrnn.backend.initializations — PsychRNN 1.0.0-alpha documentation


PsychRNN

1.0

Contents:

- Installation Guide
- API Documentation
- Getting Started

PsychRNN

- Docs »
- Module code »
- psychrnn.backend.initializations

---

# Source code for psychrnn.backend.initializations

```
from __future__ import division

import numpy as np
import tensorflow as tf
from warnings import warn

tf.compat.v1.disable_eager_execution()

[docs]class WeightInitializer(object):
    """ Base Weight Initialization class.

    Initializes biological constraints and network weights, optionally loading weights from a file or from passed in arrays.

    Keyword Arguments:
        N_in (int): The number of network inputs.
        N_rec (int): The number of recurrent units in the network.
        N_out (int): The number of network outputs.
        load_weights_path (str, optional): Path to load weights from using np.load. Weights saved at that path should be in the form saved out by :func:`psychrnn.backend.rnn.RNN.save` Default: None.
        
        input_connectivity (ndarray(dtype=float, shape=(:attr:`N_rec`, :attr:`N_in`)), optional): Connectivity mask for the input layer. 1 where connected, 0 where unconnected. Default: np.ones((:attr:`N_rec`, :attr:`N_in`)).
        rec_connectivity (ndarray(dtype=float, shape=(:attr:`N_rec`, :attr:`N_rec`)), optional): Connectivity mask for the recurrent layer. 1 where connected, 0 where unconnected. Default: np.ones((:attr:`N_rec`, :attr:`N_rec`)).
        output_connectivity (ndarray(dtype=float, shape=(:attr:`N_out`, :attr:`N_rec`)), optional): Connectivity mask for the output layer. 1 where connected, 0 where unconnected. Default: np.ones((:attr:`N_out`, :attr:`N_rec`)).
        autapses (bool, optional): If False, self connections are not allowed in N_rec, and diagonal of :data:`rec_connectivity` will be set to 0. Default: True.
        dale_ratio (float, optional): Dale's ratio, used to construct Dale_rec and Dale_out. 0 <= dale_ratio <=1 if dale_ratio should be used. ``dale_ratio * N_rec`` recurrent units will be excitatory, the rest will be inhibitory. Default: None
        
        which_rand_init (str, optional): Which random initialization to use for W_in and W_out. Will also be used for W_rec if :data:`which_rand_W_rec_init` is not passed in. Options: :func:`'const_unif' <const_unif_init>`, :func:`'const_gauss' <const_gauss_init>`, :func:`'glorot_unif' <glorot_unif_init>`, :func:`'glorot_gauss' <glorot_gauss_init>`. Default: :func:`'glorot_gauss' <glorot_gauss_init>`.
        which_rand_W_rec_init (str, optional): Which random initialization to use for W_rec. Options: :func:`'const_unif' <const_unif_init>`, :func:`'const_gauss' <const_gauss_init>`, :func:`'glorot_unif' <glorot_unif_init>`, :func:`'glorot_gauss' <glorot_gauss_init>`. Default: :data:`which_rand_init`.
        init_minval (float, optional): Used by :func:`const_unif_init` as :attr:`minval` if ``'const_unif'`` is passed in for :data:`which_rand_init` or :data:`which_rand_W_rec_init`. Default: -.1.
        init_maxval (float, optional): Used by :func:`const_unif_init` as :attr:`maxval` if ``'const_unif'`` is passed in for :data:`which_rand_init` or :data:`which_rand_W_rec_init`. Default: .1. 

        W_in (ndarray(dtype=float, shape=(:attr:`N_rec`, :attr:`N_in` )), optional): Input weights. Default: Initialized using the function indicated by :data:`which_rand_init`
        W_rec (ndarray(dtype=float, shape=(:attr:`N_rec`, :attr:`N_rec` )), optional): Recurrent weights. Default: Initialized using the function indicated by :data:`which_rand_W_rec_init`
        W_out (ndarray(dtype=float, shape=(:attr:`N_out`, :attr:`N_rec` )), optional): Output weights. Defualt: Initialized using the function indicated by :data:`which_rand_init`
        b_rec (ndarray(dtype=float, shape=(:attr:`N_rec`, )), optional): Recurrent bias. Default: np.zeros(:attr:`N_rec`)
        b_out (ndarray(dtype=float, shape=(:attr:`N_out`, )), optional): Output bias. Default: np.zeros(:attr:`N_out`)
        Dale_rec (ndarray(dtype=float, shape=(:attr:`N_rec`, :attr:`N_rec`)), optional): Diagonal matrix with ones and negative ones on the diagonal. If :data:`dale_ratio` is not ``None``, indicates whether a recurrent unit is excitatory(1) or inhibitory(-1). Default: constructed based on :data:`dale_ratio`
        Dale_out (ndarray(dtype=float, shape=(:attr:`N_rec`, :attr:`N_rec`)), optional): Diagonal matrix with ones and zeroes on the diagonal. If :data:`dale_ratio` is not ``None``, indicates whether a recurrent unit is excitatory(1) or inhibitory(0). Inhibitory neurons do not contribute to the output. Default: constructed based on :data:`dale_ratio`
        init_state (ndarray(dtype=float, shape=(1, :attr:`N_rec` )), optional): Initial state of the network's recurrent units. Default: .1 + .01 * np.random.randn(:data:`N_rec` ).

    Attributes:
        initializations (dict): Dictionary containing entries for :data:`input_connectivity`, :data:`rec_connectivity`, :data:`output_connectivity`, :data:`dale_ratio`, :data:`Dale_rec`, :data:`Dale_out`, :data:`W_in`, :data:`W_rec`, :data:`W_out`, :data:`b_rec`, :data:`b_out`, and :data:`init_state`.
    
    """


    def __init__(self, **kwargs):

        # ----------------------------------
        # Required parameters
        # ----------------------------------
        self.load_weights_path = kwargs.get('load_weights_path', None)
        N_in = self.N_in = kwargs.get('N_in')
        N_rec = self.N_rec = kwargs.get('N_rec')
        N_out = self.N_out = kwargs.get('N_out')
        self.autapses = kwargs.get('autapses', True)

        self.initializations = dict()

        if self.load_weights_path is not None:
            # ----------------------------------
            # Load saved weights
            # ----------------------------------
            self.initializations = dict(np.load(self.load_weights_path, allow_pickle = True))
            if type(self.initializations['dale_ratio']) == np.ndarray:
                self.initializations['dale_ratio'] = self.initializations['dale_ratio'].item()

        else:
            if kwargs.get('W_rec', None) is None and type(self).__name__=='WeightInitializer':
                warn("This network may not train since the eigenvalues of W_rec are not regulated in any way.")

            # ----------------------------------
            # Optional Parameters
            # ----------------------------------
            self.rand_init = kwargs.get('which_rand_init', 'glorot_gauss')
            self.rand_W_rec_init = self.get_rand_init_func(kwargs.get('which_rand_W_rec_init', self.rand_init))
            self.rand_init = self.get_rand_init_func(self.rand_init)
            if self.rand_init == self.const_unif_init or self.rand_W_rec_init == self.const_unif_init:
                self.init_minval = kwargs.get('init_minval', -.1)
                self.init_maxval = kwargs.get('init_maxval', .1)

            # ----------------------------------
            # Biological Constraints
            # ----------------------------------

            # Connectivity constraints
            self.initializations['input_connectivity'] = kwargs.get('input_connectivity',np.ones([N_rec, N_in]))
            assert(self.initializations['input_connectivity'].shape == (N_rec, N_in))
            self.initializations['rec_connectivity'] = kwargs.get('rec_connectivity',np.ones([N_rec, N_rec]))
            assert(self.initializations['rec_connectivity'].shape == (N_rec, N_rec))
            self.initializations['output_connectivity'] = kwargs.get('output_connectivity', np.ones([N_out, N_rec]))
            assert(self.initializations['output_connectivity'].shape == (N_out, N_rec))
            
            # Autapses constraint
            if not self.autapses:
                self.initializations['rec_connectivity'][np.eye(N_rec) == 1] = 0

            # Dale's constraint
            self.initializations['dale_ratio'] = dale_ratio = kwargs.get('dale_ratio', None)
            if type(self.initializations['dale_ratio']) == np.ndarray:
                self.initializations['dale_ratio'] = dale_ratio = self.initializations['dale_ratio'].item()
            if dale_ratio is not None and (dale_ratio <0 or dale_ratio > 1):
                print("Need 0 <= dale_ratio <= 1. dale_ratio was: " + str(dale_ratio))
                raise
            dale_vec = np.ones(N_rec)
            if dale_ratio is not None:
                dale_vec[int(dale_ratio * N_rec):] = -1
                dale_rec = np.diag(dale_vec)
                dale_vec[int(dale_ratio * N_rec):] = 0
                dale_out = np.diag(dale_vec)
            else:
                dale_rec = np.diag(dale_vec)
                dale_out = np.diag(dale_vec)
            self.initializations['Dale_rec'] = kwargs.get('Dale_rec', dale_rec)
            assert(self.initializations['Dale_rec'].shape == (N_rec, N_rec))
            self.initializations['Dale_out'] = kwargs.get('Dale_out', dale_rec)
            assert(self.initializations['Dale_out'].shape == (N_rec, N_rec))

            # ----------------------------------
            # Default initializations / optional loading from params
            # ----------------------------------

            self.initializations['W_in'] = kwargs.get('W_in', self.rand_init(self.initializations['input_connectivity']))
            assert(self.initializations['W_in'].shape == (N_rec, N_in))
            self.initializations['W_out'] = kwargs.get('W_out', self.rand_init(self.initializations['output_connectivity']))
            assert(self.initializations['W_out'].shape == (N_out, N_rec))
            self.initializations['W_rec'] = kwargs.get('W_rec', self.rand_W_rec_init(self.initializations['rec_connectivity']))
            assert(self.initializations['W_rec'].shape == (N_rec, N_rec))

            self.initializations['b_rec'] = kwargs.get('b_rec',np.zeros(N_rec))
            assert(self.initializations['b_rec'].shape == (N_rec,))
            self.initializations['b_out'] = kwargs.get('b_out',np.zeros(N_out))
            assert(self.initializations['b_out'].shape == (N_out,))

            self.initializations['init_state'] = kwargs.get('init_state', .1 + .01 * np.random.randn(N_rec))
            assert(self.initializations['init_state'].size == N_rec)

        return

[docs]    def get_rand_init_func(self, which_rand_init):
        """Maps initialization function names (strings) to generating functions.

        Arguments:
            which_rand_init (str): Maps to ``[which_rand_init]_init``. Options are :func:`'const_unif' <const_unif_init>`, :func:`'const_gauss' <const_gauss_init>`, :func:`'glorot_unif' <glorot_unif_init>`, :func:`'glorot_gauss' <glorot_gauss_init>`.

        Returns:
            function: ``self.[which_rand_init]_init``

        """
        mapping = {
            'const_unif': self.const_unif_init,
            'const_gauss': self.const_gauss_init,
            'glorot_unif': self.glorot_unif_init,
            'glorot_gauss': self.glorot_gauss_init}
        return mapping[which_rand_init]


[docs]    def const_gauss_init(self, connectivity):
        """ Initialize ndarray of shape :data:`connectivity` with values from a normal distribution.

        Arguments:
            connectivity (ndarray): 1 where connected, 0 where unconnected.

        Returns:
            ndarray(dtype=float, shape=connectivity.shape)

        """
        return np.random.randn(connectivity.shape[0], connectivity.shape[1])


[docs]    def const_unif_init(self, connectivity):
        """ Initialize ndarray of shape :data:`connectivity` with values uniform distribution with minimum :data:`init_minval` and maximum :data:`init_maxval` as set in :class:`WeightInitializer`.

        Arguments:
            connectivity (ndarray): 1 where connected, 0 where unconnected.

        Returns:
            ndarray(dtype=float, shape=connectivity.shape)

        """
        minval = self.init_minval
        maxval = self.init_maxval
        return (maxval-minval) * np.random.rand(connectivity.shape[0], connectivity.shape[1]) + minval


[docs]    def glorot_unif_init(self, connectivity):
        """ Initialize ndarray of shape :data:`connectivity` with values from a glorot uniform distribution.

        Draws samples from a uniform distribution within [-limit, limit] where `limit`
        is `sqrt(6 / (fan_in + fan_out))` where `fan_in` is the number of input units and `fan_out` is the number of output units. Respects the :data:`connectivity` matrix.

        Arguments:
            connectivity (ndarray): 1 where connected, 0 where unconnected.

        Returns:
            ndarray(dtype=float, shape=connectivity.shape)

        """


        init = np.zeros(connectivity.shape)
        fan_in = np.sum(connectivity, axis = 1)
        init += np.tile(fan_in, (connectivity.shape[1],1)).T
        fan_out = np.sum(connectivity, axis = 0)
        init += np.tile(fan_out, (connectivity.shape[0],1))
        return np.random.uniform(-np.sqrt(6/init), np.sqrt(6/init))


[docs]    def glorot_gauss_init(self, connectivity):
        """ Initialize ndarray of shape :data:`connectivity` with values from a glorot normal distribution.

        Draws samples from a normal distribution centered on 0 with `stddev
        = sqrt(2 / (fan_in + fan_out))` where `fan_in` is the number of input units and `fan_out` is the number of output units. Respects the :data:`connectivity` matrix.

        Arguments:
            connectivity (ndarray): 1 where connected, 0 where unconnected.

        Returns:
            ndarray(dtype=float, shape=connectivity.shape)

        """

        init = np.zeros(connectivity.shape)
        fan_in = np.sum(connectivity, axis = 1)
        init += np.tile(fan_in, (connectivity.shape[1],1)).T
        fan_out = np.sum(connectivity, axis = 0)
        init += np.tile(fan_out, (connectivity.shape[0],1))
        return np.random.normal(0, np.sqrt(2/init))


[docs]    def get_dale_ratio(self):
        """ Returns the dale_ratio.

        :math:`0 \\leq dale\\_ratio \\leq 1` if dale_ratio should be used, dale_ratio = None otherwise. ``dale_ratio * N_rec`` recurrent units will be excitatory, the rest will be inhibitory.

        Returns:
            float: Dale ratio, None if no dale ratio is set.
        """

        return self.initializations['dale_ratio']


[docs]    def get(self, tensor_name):
        """ Get :data:`tensor_name` from :attr:`initializations` as a Tensor.

        Arguments:
            tensor_name (str): The name of the tensor to get. See :attr:`initializations` for options.

        Returns:
            Tensor object

        """

        return tf.compat.v1.constant_initializer(self.initializations[tensor_name])


[docs]    def save(self, save_path):
        """ Save :attr:`initializations` to :data:`save_path`.

        Arguments:
            save_path (str): File path for saving the initializations. The .npz extension will be appended if not already provided.
        """

        np.savez(save_path, **self.initializations)
        return


[docs]    def balance_dale_ratio(self):
        """ If dale_ratio is not None, balances :attr:`initializations['W_rec'] <initializations>` 's excitatory and inhibitory weights so the network will train. 
        """
        dale_ratio = self.get_dale_ratio()
        if dale_ratio is not None:
            dale_vec = np.ones(self.N_rec)
            dale_vec[int(dale_ratio * self.N_rec):] = dale_ratio/(1-dale_ratio)
            dale_rec = np.diag(dale_vec) / np.linalg.norm(np.matmul(self.initializations['rec_connectivity'], np.diag(dale_vec)), axis=1)[:,np.newaxis]
            self.initializations['W_rec'] = np.matmul(self.initializations['W_rec'], dale_rec)
        return


[docs]class GaussianSpectralRadius(WeightInitializer):
    """Generate random gaussian weights with specified spectral radius.

    If Dale is set, balances the random gaussian weights between excitatory and inhibitory using :func:`balance_dale_ratio` before applying the specified spectral radius.

    Keyword Args:
       spec_rad (float, optional): The spectral radius to initialize W_rec with. Default: 1.1.

    Other Keyword Args:
        See :class:`~psychrnn.backend.initializations.WeightInitializer` for details.
    """

    def __init__(self, **kwargs):

        super(GaussianSpectralRadius, self).__init__(**kwargs)

        self.spec_rad = kwargs.get('spec_rad', 1.1)

        self.initializations['W_rec']  = np.random.randn(self.N_rec, self.N_rec)

        # balance weights for dale ratio training to proceed normally
        self.balance_dale_ratio()

        self.initializations['W_rec'] = self.spec_rad * self.initializations['W_rec'] / np.max(np.abs(np.linalg.eig(self.initializations['W_rec'])[0]))

        return


[docs]class AlphaIdentity(WeightInitializer):
    '''Generate recurrent weights :math:`w(i,i) = alpha`, :math:`w(i,j) = 0` where :math:`i \\neq j`.

    If Dale is set, balances the alpha excitatory and inhibitory weights using :func:`~psychrnn.backend.initializations.WeightInitializer.balance_dale_ratio`, so w(i,i) will not be exactly equal to alpha.

    Keyword Args:
       alpha (float): The value of alpha to set w(i,i) to in W_rec.

    Other Keyword Args:
        See :class:`WeightInitializer` for details.
    '''

    def __init__(self, **kwargs):

        super(AlphaIdentity, self).__init__(**kwargs)

        self.alpha = kwargs.get('alpha')

        self.initializations['W_rec'] = np.eye(self.N_rec) * self.alpha
        
        # balance weights for dale ratio training to proceed normally
        self.balance_dale_ratio()

        return
```

---

© Copyright 2020, Authors redacted for double-blind review

Built with Sphinx using a theme provided by Read the Docs.
