# Supplementary material for: PsychRNN: An Accessible and Flexible Python Package for Training Recurrent Neural Network Models on Cognitive Tasks
Source: eNeuro. 2021 Jan 5;8(1):ENEURO.0427-20.2020. doi: 10.1523/ENEURO.0427-20.2020 (PMC7814477; doi:10.1523/ENEURO.0427-20.2020)
Supplement: Extended Data 1 — PsychRNN package and documentation. Download Extended Data 1, ZIP file. [file enu-eN-OTM-0427-20-s06.zip › PsychRNN Extended Data 1/docs/_build/html/_modules/psychrnn/backend/models/basic.html]

  


psychrnn.backend.models.basic — PsychRNN 1.0.0-alpha documentation


PsychRNN

1.0

Contents:

- Installation Guide
- API Documentation
- Getting Started

PsychRNN

- Docs »
- Module code »
- psychrnn.backend.models.basic

---

# Source code for psychrnn.backend.models.basic

```
from __future__ import division

from psychrnn.backend.rnn import RNN
import tensorflow as tf
tf.compat.v1.disable_eager_execution()

[docs]class Basic(RNN):
    """ The basic continuous time recurrent neural network model.

    Basic implementation of :class:`psychrnn.backend.rnn.RNN` with a simple RNN, enabling biological constraints.

    Args:
       params (dict): See :class:`psychrnn.backend.rnn.RNN` for details.

    """

[docs]    def recurrent_timestep(self, rnn_in, state):
        """ Recurrent time step.

        Given input and previous state, outputs the next state of the network.

        Arguments:
            rnn_in (*tf.Tensor(dtype=float, shape=(?*, :attr:`N_in` *))*): Input to the rnn at a certain time point.
            state (*tf.Tensor(dtype=float, shape=(* :attr:`N_batch` , :attr:`N_rec` *))*): State of network at previous time point.

        Returns:
            new_state (*tf.Tensor(dtype=float, shape=(* :attr:`N_batch` , :attr:`N_rec` *))*): New state of the network.

        """

        new_state = ((1-self.alpha) * state) \
                    + self.alpha * (
                        tf.matmul(
                            self.transfer_function(state),
                            self.get_effective_W_rec(),
                            transpose_b=True, name="1")
                        + tf.matmul(
                            rnn_in,
                            self.get_effective_W_in(),
                            transpose_b=True, name="2")
                        + self.b_rec)\
                    + tf.sqrt(2.0 * self.alpha * self.rec_noise * self.rec_noise)\
                      * tf.random.normal(tf.shape(input=state), mean=0.0, stddev=1.0)

        return new_state


[docs]    def output_timestep(self, state):
        """Returns the output node activity for a given timestep.

        Arguments:
            state (*tf.Tensor(dtype=float, shape=(* :attr:`N_batch` , :attr:`N_rec` *))*): State of network at a given timepoint for each trial in the batch.

        Returns:
            output (*tf.Tensor(dtype=float, shape=(* :attr:`N_batch` , :attr:`N_out` *))*): Output of the network at a given timepoint for each trial in the batch.

        """
     
        output = tf.matmul(self.transfer_function(state),
                                self.get_effective_W_out(), transpose_b=True, name="3") \
                    + self.b_out
     
        return output


[docs]    def forward_pass(self):

        """ Run the RNN on a batch of task inputs.

        Iterates over timesteps, running the :func:`recurrent_timestep` and :func:`output_timestep`

        Implements :func:`psychrnn.backend.rnn.RNN.forward_pass`.

        Returns:
            tuple:
            * **predictions** (*tf.Tensor(*:attr:`N_batch`, :attr:`N_steps`, :attr:`N_out` *))*) -- Network output on inputs found in self.x within the tf network.
            * **states** (*tf.Tensor(*:attr:`N_batch`, :attr:`N_steps`, :attr:`N_rec` *))*) -- State variable values over the course of the trials found in self.x within the tf network.

        """

        rnn_inputs = tf.unstack(self.x, axis=1)
        state = self.init_state
        rnn_outputs = []
        rnn_states = []
        for rnn_input in rnn_inputs:
            state = self.recurrent_timestep(rnn_input, state)
            output = self.output_timestep(state)
            rnn_outputs.append(output)
            rnn_states.append(state)
        return tf.transpose(a=rnn_outputs, perm=[1, 0, 2]), tf.transpose(a=rnn_states, perm=[1, 0, 2])


[docs]class BasicScan(Basic):
    """ The basic continuous time recurrent neural network model implemented with `tf.scan <https://www.tensorflow.org/api_docs/python/tf/scan>`_ .

    Produces the same results as :class:`Basic`, with possible differences in execution time.

    Args:
       params (dict): See :class:`psychrnn.backend.rnn.RNN` for details.

    """

[docs]    def recurrent_timestep(self, state, rnn_in):
        """ Wrapper function for :func:`psychrnn.backend.models.basic.Basic.recurrent_timestep`. 

        Arguments:
            state (*tf.Tensor(dtype=float, shape=(* :attr:`N_batch` , :attr:`N_rec` *))*): State of network at previous time point.
            rnn_in (*tf.Tensor(dtype=float, shape=(?*, :attr:`N_in` *))*): Input to the rnn at a certain time point.
 
        Returns:
            new_state (*tf.Tensor(dtype=float, shape=(* :attr:`N_batch` , :attr:`N_rec` *))*): New state of the network.

        """

        return super(BasicScan, self).recurrent_timestep(rnn_in, state)


[docs]    def output_timestep(self, dummy, state):
        """ Wrapper function for :func:`psychrnn.backend.models.basic.Basic.output_timestep`.

        Includes additional dummy argument to facilitate `tf.scan <https://www.tensorflow.org/api_docs/python/tf/scan>`_.

        Arguments:
            dummy: Dummy variable provided by tf.scan. Not actually used by the function.
            state (*tf.Tensor(dtype=float, shape=(* :attr:`N_batch` , :attr:`N_rec` *))*): State of network at a given timepoint for each trial in the batch.

        Returns:
            output (*tf.Tensor(dtype=float, shape=(* :attr:`N_batch` , :attr:`N_out` *))*): Output of the network at a given timepoint for each trial in the batch.

        """
        return super(BasicScan, self).output_timestep(state)


[docs]    def forward_pass(self):
        """ Run the RNN on a batch of task inputs.

        Iterates over timesteps, running the :func:`recurrent_timestep` and :func:`output_timestep`

        Implements :func:`psychrnn.backend.rnn.RNN.forward_pass`.

        Returns:
            tuple:
            * **predictions** (*tf.Tensor(*:attr:`N_batch`, :attr:`N_steps`, :attr:`N_out` *))*) -- Network output on inputs found in self.x within the tf network.
            * **states** (*tf.Tensor(*:attr:`N_batch`, :attr:`N_steps`, :attr:`N_rec` *))*) -- State variable values over the course of the trials found in self.x within the tf network.

        """

        state = self.init_state
        rnn_states = \
            tf.scan(
                self.recurrent_timestep,
                tf.transpose(a=self.x, perm=[1, 0, 2]),
                initializer=state,
                parallel_iterations=1)
        rnn_outputs = \
            tf.scan(
                self.output_timestep,
                rnn_states,
                initializer=tf.zeros([self.N_batch, self.N_out]),
                parallel_iterations=1)
        return tf.transpose(a=rnn_outputs, perm=[1, 0, 2]), tf.transpose(a=rnn_states, perm=[1, 0, 2])
```

---

© Copyright 2020, Authors redacted for double-blind review

Built with Sphinx using a theme provided by Read the Docs.
