# Supplementary material for: PsychRNN: An Accessible and Flexible Python Package for Training Recurrent Neural Network Models on Cognitive Tasks
Source: eNeuro. 2021 Jan 5;8(1):ENEURO.0427-20.2020. doi: 10.1523/ENEURO.0427-20.2020 (PMC7814477; doi:10.1523/ENEURO.0427-20.2020)
Supplement: Extended Data 1 — PsychRNN package and documentation. Download Extended Data 1, ZIP file. [file enu-eN-OTM-0427-20-s06.zip › PsychRNN Extended Data 1/docs/_build/html/_modules/psychrnn/backend/models/lstm.html]

  


psychrnn.backend.models.lstm — PsychRNN 1.0.0-alpha documentation


PsychRNN

1.0

Contents:

- Installation Guide
- API Documentation
- Getting Started

PsychRNN

- Docs »
- Module code »
- psychrnn.backend.models.lstm

---

# Source code for psychrnn.backend.models.lstm

```
from __future__ import division

from psychrnn.backend.rnn import RNN
import tensorflow as tf

tf.compat.v1.disable_eager_execution()

[docs]class LSTM(RNN):
    """ LSTM (Long Short Term Memory) recurrent network model

    LSTM implementation of :class:`psychrnn.backend.rnn.RNN`. Because LSTM is structured differently from the basic RNN, biological constraints such as dale's, autapses, and connectivity are not enabled.

    Args:
       params (dict): See :class:`psychrnn.backend.rnn.RNN` for details.

    """

    def __init__(self, params):
        # ----------------------------------
        # Call RNN constructor
        # ----------------------------------
        super(LSTM, self).__init__(params)

        # ----------------------------------
        # Add new variables for gates
        # TODO better LSTM initialization
        # ----------------------------------
        self.N_concat = self.N_in + self.N_rec

        self.init_hidden_initializer = tf.compat.v1.random_normal_initializer(mean=0, stddev=0.1)
        self.init_cell_initializer = tf.compat.v1.random_normal_initializer(mean=0, stddev=0.1)

        self.W_f_initializer = tf.compat.v1.random_normal_initializer(mean=0, stddev=0.1)
        self.W_i_initializer = tf.compat.v1.random_normal_initializer(mean=0, stddev=0.1)
        self.W_c_initializer = tf.compat.v1.random_normal_initializer(mean=0, stddev=0.1)
        self.W_o_initializer = tf.compat.v1.random_normal_initializer(mean=0, stddev=0.1)

        self.b_f_initializer = tf.compat.v1.constant_initializer(1.0)
        self.b_i_initializer = tf.compat.v1.constant_initializer(1.0)
        self.b_c_initializer = tf.compat.v1.constant_initializer(1.0)
        self.b_o_initializer = tf.compat.v1.constant_initializer(1.0)

        # ----------------------------------
        # TensorFlow initializations
        # ----------------------------------
        with tf.compat.v1.variable_scope(self.name) as scope:
            self.init_hidden = tf.compat.v1.get_variable('init_hidden', [self.N_batch, self.N_rec],
                                               initializer=self.init_hidden_initializer,
                                                trainable=True)
            self.init_cell = tf.compat.v1.get_variable('init_cell', [self.N_batch, self.N_rec],
                                             initializer=self.init_cell_initializer,
                                            trainable=True)

            self.W_f = tf.compat.v1.get_variable('W_f', [self.N_concat, self.N_rec],
                                            initializer=self.W_f_initializer,
                                            trainable=True)
            self.W_i = tf.compat.v1.get_variable('W_i', [self.N_concat, self.N_rec],
                                            initializer=self.W_i_initializer,
                                            trainable=True)
            self.W_c = tf.compat.v1.get_variable('W_c', [self.N_concat, self.N_rec],
                                            initializer=self.W_c_initializer,
                                            trainable=True)
            self.W_o = tf.compat.v1.get_variable('W_o', [self.N_concat, self.N_rec],
                                            initializer=self.W_o_initializer,
                                            trainable=True)

            self.b_f = tf.compat.v1.get_variable('b_f', [self.N_rec], initializer=self.b_f_initializer,
                                         trainable=True)
            self.b_i = tf.compat.v1.get_variable('b_i', [self.N_rec], initializer=self.b_i_initializer,
                                       trainable=True)
            self.b_c = tf.compat.v1.get_variable('b_c', [self.N_rec], initializer=self.b_c_initializer,
                                       trainable=True)
            self.b_o = tf.compat.v1.get_variable('b_o', [self.N_rec], initializer=self.b_o_initializer,
                                       trainable=True)

[docs]    def recurrent_timestep(self, rnn_in, hidden, cell):
        """ Recurrent time step.

        Given input and previous state, outputs the next state of the network.

        Arguments:
            rnn_in (*tf.Tensor(dtype=float, shape=(?*, :attr:`N_in` *))*): Input to the rnn at a certain time point.
            hidden (*tf.Tensor(dtype=float, shape=(* :attr:`N_batch` , :attr:`N_rec` *))*): Hidden units state of network at previous time point.
            cell (*tf.Tensor(dtype=float, shape=(* :attr:`N_batch` , :attr:`N_rec` *))*): Cell state of the network at previous time point.

        Returns:
            tuple:
            * **new_hidden** (*tf.Tensor(dtype=float, shape=(* :attr:`N_batch` , :attr:`N_rec` *))*) -- New hidden unit state of the network.
            * **new_cell** (*tf.Tensor(dtype=float, shape=(* :attr:`N_batch` , :attr:`N_rec` *))*) -- New cell state of the network.
        """

        f = tf.nn.sigmoid(tf.matmul(tf.concat([hidden, rnn_in], 1), self.W_f)
                               + self.b_f)

        i = tf.nn.sigmoid(tf.matmul(tf.concat([hidden, rnn_in], 1), self.W_i)
                               + self.b_i)

        c = tf.nn.tanh(tf.matmul(tf.concat([hidden, rnn_in], 1), self.W_c)
                               + self.b_c)

        o = tf.nn.sigmoid(tf.matmul(tf.concat([hidden, rnn_in], 1), self.W_o)
                          + self.b_o)

        new_cell = f * cell + i * c

        new_hidden = o * tf.nn.sigmoid(new_cell)

        return new_hidden, new_cell


[docs]    def output_timestep(self, hidden):
        """Returns the output node activity for a given timestep.

        Arguments:
            hidden (*tf.Tensor(dtype=float, shape=(* :attr:`N_batch` , :attr:`N_rec` *))*): Hidden units of network at a given timepoint for each trial in the batch.

        Returns:
            output (*tf.Tensor(dtype=float, shape=(* :attr:`N_batch` , :attr:`N_out` *))*): Output of the network at a given timepoint for each trial in the batch.

        """

        output = tf.matmul(hidden, self.W_out, transpose_b=True) + self.b_out

        return output


[docs]    def forward_pass(self):
        """ Run the LSTM on a batch of task inputs.

        Iterates over timesteps, running the :func:`recurrent_timestep` and :func:`output_timestep`

        Implements :func:`psychrnn.backend.rnn.RNN.forward_pass`.

        Returns:
            tuple:
            * **predictions** (*tf.Tensor(*:attr:`N_batch`, :attr:`N_steps`, :attr:`N_out` *))*) -- Network output on inputs found in self.x within the tf network.
            * **hidden** (*tf.Tensor(*:attr:`N_batch`, :attr:`N_steps`, :attr:`N_rec` *))*) -- Hidden unit values over the course of the trials found in self.x within the tf network.

        """

        rnn_inputs = tf.unstack(self.x, axis=1)
        hidden = self.init_hidden
        cell = self.init_cell
        rnn_outputs = []
        rnn_states = []
        for rnn_input in rnn_inputs:
            hidden, cell = self.recurrent_timestep(rnn_input, hidden, cell)
            output = self.output_timestep(hidden)
            rnn_outputs.append(output)
            rnn_states.append(hidden)

        return tf.transpose(a=rnn_outputs, perm=[1, 0, 2]), tf.transpose(a=rnn_states, perm=[1, 0, 2])
```

---

© Copyright 2020, Authors redacted for double-blind review

Built with Sphinx using a theme provided by Read the Docs.
